# Infrared Photon Pair-Production in Ligand-Sensitized Lanthanide Nanocrystals

**DOI:** 10.3389/fchem.2020.579942

**Published:** 2020-11-04

**Authors:** Peter Agbo, Jacob S. Kanady, Rebecca J. Abergel

**Affiliations:** ^1^Chemical Sciences Division, Lawrence Berkeley National Laboratory, Berkeley, CA, United States; ^2^Department of Chemistry, University of California, Berkeley, Berkeley, CA, United States; ^3^Department of Nuclear Engineering, University of California, Berkeley, Berkeley, CA, United States

**Keywords:** sensitization, lanthanide, two-photon, downconversion, energy transfer

## Abstract

This report details spectroscopic characterizations of rare-earth, core-shell nanoparticles decorated with the *f*-element chelator 3,4,3-LI(1,2-HOPO). Evidence of photon downconversion is corroborated through detailed power dependence measurements, which suggest two-photon decay paths are active in these materials, albeit only representing a minority contribution of the sum luminescence, with emission being dominated by normal, Stokes' shifted fluorescence. Specifically, ultraviolet ligand photosensitization of Nd^3+^ ions in a NaGdF_4_ host shell results in energy transfer to a Nd^3+^/Yb^3+^-doped NaGdF_4_ nanoparticle core. The population and subsequent decay of core, Yb^3+^ ^2^*F*_5/2_ states result in a spectral shift of 620 nm, manifested in a NIR emission displaying luminescence profiles diagnostic of Yb^3+^ and Nd^3+^ excited state decays. Emphasis is placed on the generality of this material architecture for realizing ligand-pumped, multi-photon downconversion, with the Nd^3+^/Yb^3+^ system presented here functioning as a working prototype for a design principle that may be readily extended to other lanthanide pairs.

## Introduction

Broadening the spectral bandwidth of conventional photovoltaics remains one of the chief avenues for generating photocurrent at the detailed-balance limit described by Shockley and Queisser (1961). Realization of such advanced single-junction devices mandates the address of spectral mismatching between semiconductor absorption profiles and the terrestrial solar spectrum. Despite a sizable body of scholarship devoted to light upconversion (UC), comparatively little work has addressed the challenge of ultraviolet (UV) downconversion (DC) toward the low-energy visible and near-infrared (NIR) regimes, where the photocurrent response for bulk Si is highest. Currently, the practical implementation of downconverting lanthanide (Ln) materials in solar arrays has largely been limited by the low absorption intensities typical of *f-f* transitions (~ 10 M^−1^ cm^−1^), resulting in low external quantum efficiencies (Shavaleev et al., 2005; Werts, 2005; Zhang et al., 2007; Charbonnière et al., 2008; Moore et al., 2008, 2009; Van Der Ende et al., 2009; Bünzli and Eliseeva, 2010; Janssens et al., 2011; Li S. et al., 2012; Gauthier et al., 2013; Bünzli and Chauvin, 2014; Li S. W. et al., 2014; Binnemans, 2015; Irfanullah et al., 2015, 2016; Goetz et al., 2016; Song et al., 2016). Methods previously explored include the relaxation of Laporte selection rules through the embedding of Ln ions in low-symmetry crystal hosts, and the photosensitization of *f*-states through the use of either ligand-to-metal charge transfer transitions in transition metal ions, or inter-band (*d-f* ) charge transfer in Ln such as Ce^3+^ or Eu^2+^ (Sun et al., 2017). By contrast, the possibility of assisting spectral conversion yields in Ln nanoparticles (NPs) through the use of organic ligands remains a relatively novel method of enhancing f-block NP light absorption (Garfield et al., 2018). Recent work by Meijerink et al. described a dye-sensitized NP system showing successful Pr^3+^/Yb^3+^ energy transfer, but excluded explicit proof of two-photon production through power dependence or quantum yield determinations (Wang and Meijerink, 2018). As a complement to an earlier patent application disclosure (Agbo and Abergel, 2017), this report relates the concept of UV-NIR nanocrystals featuring the hydroxypyridinone-derived, metal chelator 3,4,3-LI(1,2-HOPO) (**343**) as a UV photosensitizer of NaGd_1−x−y_Nd_x_Yb_y_F_4_ | NaGd_1−x_Nd_x_F_4_ core|shell NPs. Our previous work demonstrated the utility of sensitizing Ln excited states in nanocrystals through energy transfer from the **343** triplet state following UV ligand absorption. Aside from a demonstration of dramatic wavelength shifts from the UV to the NIR, the material described here achieves this spectral transformation through partial utilization of decay channels that permit the production of two NIR photons per UV photon absorbed, as illustrated by a detailed power dependence study.

## Results and Discussion

**Figure 1:**
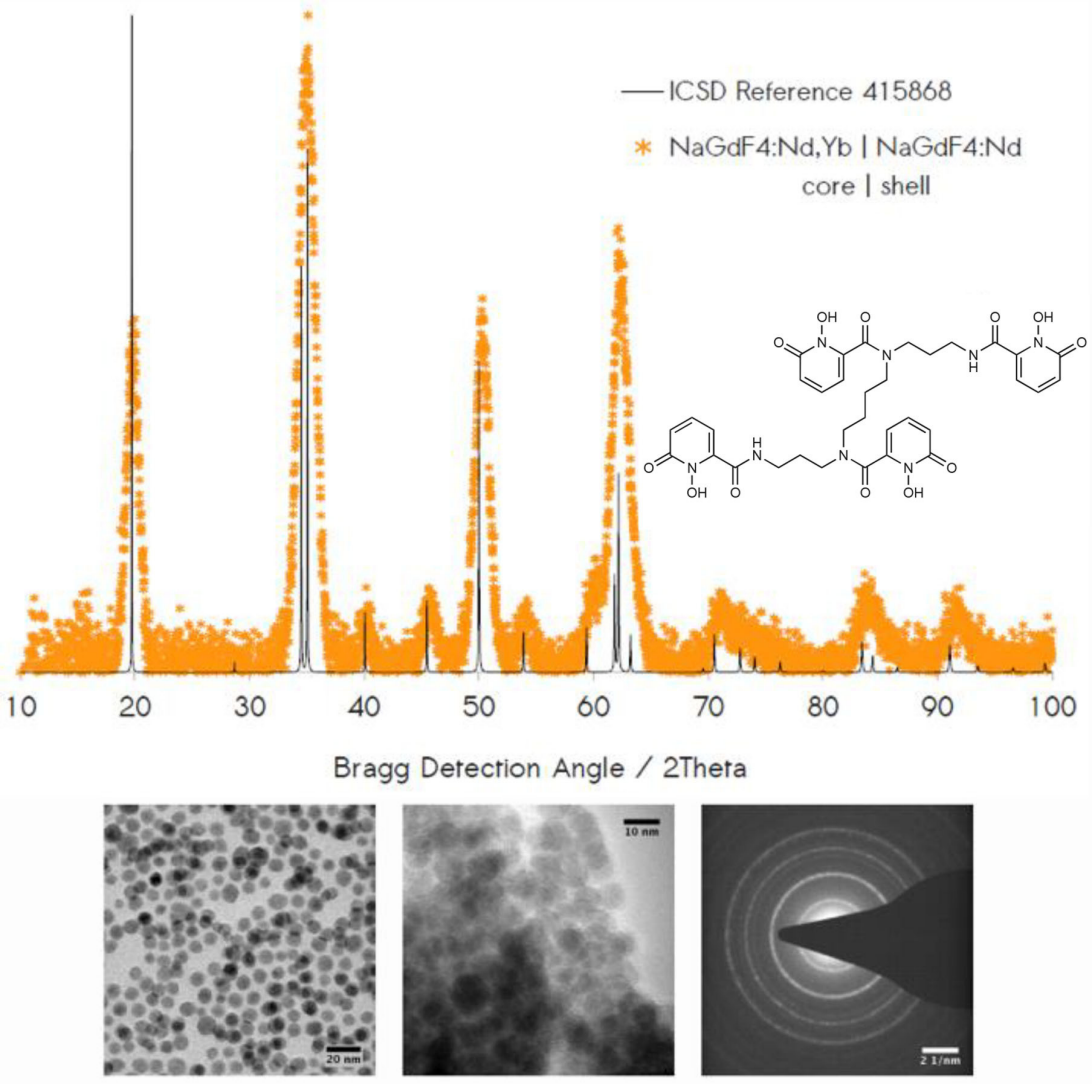
Top: Powder XRD data confirm colloidal synthesis of the hexagonal NaGdF_4_:Nd,Yb | NaGdF_4_:Nd. Inset: **343** surface chelator. Bottom: TEM reveals monodisperse nanocrystals of 10 nm in diameter in cases of unmodified (left) and **343**-chelated (center) samples. Electron diffraction (right) provides evidence of dominant Bragg diffraction from lattice planes of hkl indices: (100), (110) + (101), (201), and (300) + (121).

**Figure 2:**
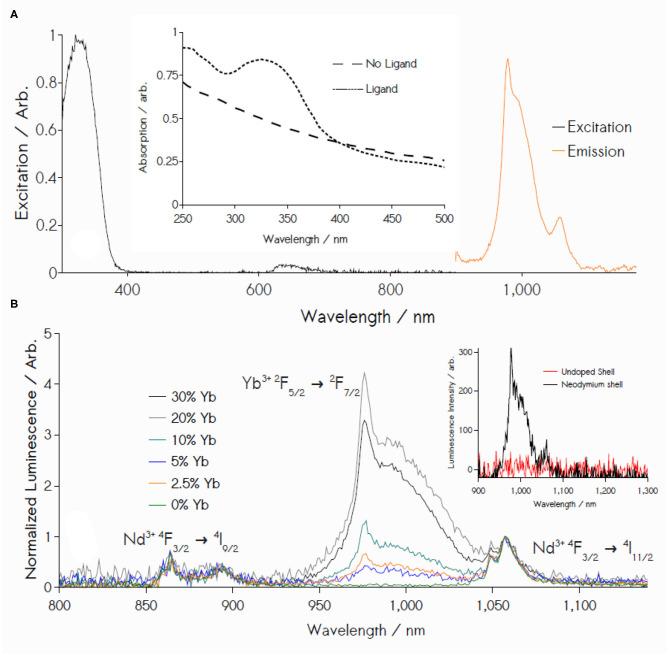
**(A)** *Black:* Excitation spectrum of 3% Nd^3+^/20% Yb^3+^-doped NPs monitoring 980 nm Yb^3+^ emission. *Orange trace:* NIR emission under 355 nm excitation. Inset: UV-vis absorption in ethanol. **(B)** Luminescence from Nd^3+^, Yb^3+^ core-shell NPs upon excitation at 355 nm, showing increasing Yb^3+^ emission (normalized relative to Nd^3+^ emission at 1059 nm) as Yb^3+^ content increases (0 to 20 mol %). Nd^3+^ content is held constant at 3 mol %. Inset: Removal of Nd^3+^ from the shell blocks energy transfer to terminal Yb^3+^ acceptor ions, arresting photoemission.

Preparation of these core-shell structures proceeded through the stepwise synthesis of doubly-doped Nd^3+^/Yb^3+^ nanocrystal cores from Ln acetate precursors (Wang et al., 2014), followed by a second synthetic round using only Nd^3+^ dopant to yield the corresponding NaGd_1−x−y_Nd_x_Yb_y_F_4_ | NaGd_1−x_Nd_x_F_4_ NPs. Successful synthesis was inferred from the results of transmission electron microscopy (TEM) and powder x-ray diffraction (XRD). Diffraction showed persistence of a single crystal phase consistent with the Bragg diffraction lines expected for the hexagonal-form, β-NaGdF_4_ (Figure 1). NP morphologies interrogated through TEM revealed monodisperse nanocrystals roughly 10 nm in diameter. High resolution imaging and electron diffraction permitted resolution of lattice plane spacings consistent with those observed in NaGdF_4_ hosts (Supplementary Figures 1–5). Modification of NP surfaces with **343** was found to promote aggregation among NPs (Supplementary Figures 1–4), consistent with earlier observations (Agbo and Abergel, 2016; Agbo et al., 2016). and was also demonstrated by UV-vis absorption (Figure 2A, inset). Absorption measurements of the unmodified particles are characterized solely by Rayleigh scatter. **343** surface chelation of these NPs results in the evolution of a broad π → π^*^ (ε~17, 000*M*^−1^*cm*^−1^) transition between ca. 300 and 360 nm, with a maximum at 325 nm (Abergel et al., 2009).

NP luminescence reveals a ligand-sensitized emission in the NIR that is a composite of Yb^3+^ (^2^*F*_5/2_ → ^2^*F*_7/2_) and Nd^3+^ (^4^*F*_3/2_ → ^4^*I*_J=9/2,11/2_) transitions (Figure 2B). Specifically, excitation spectra of these materials indicate their irradiation between ca. 300 and 360 nm results in an IR emission representing a superposition of Yb^3+^ radiative decay and peaks diagnostic of Nd^3+^ transitions at 860 nm (^4^*F*_3/2_ → ^4^*I*_9/2_) and 1059 nm (^4^*F*_3/2_ → ^4^*I*_11/2_). Varying Yb^3+^ content results in an increased Yb^3+^ luminescence until a 20% dopant concentration is reached (Figure 2B) (Lakshminarayana et al., 2008a; Li L. et al., 2012; Wang et al., 2015), which was therefore used for all subsequent measurements in this study.

**Figure 3:**
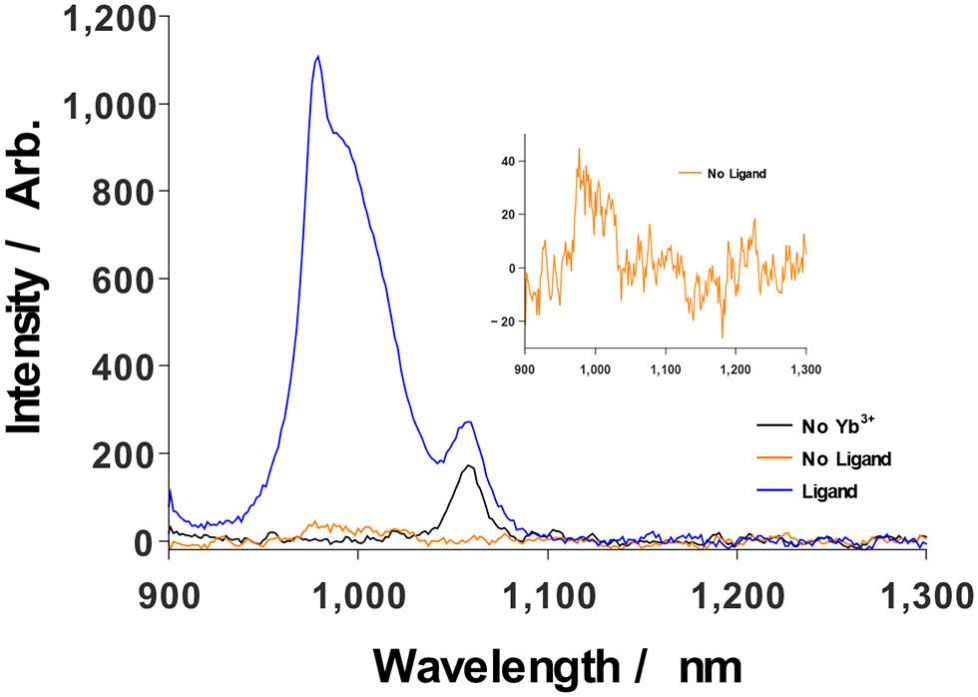
Ligand sensitization significantly enhances Yb^3+^ luminescence upon excitation at 350 nm (blue), relative to the case of NP illumination in the absence of photosensitization by **343** (orange, inset). Sensitized NPs without Yb^3+^ only show Nd^3+^ emission at 1057 nm (black).

These results are consistent with a deliberate nanocrystal design where **343** functions as a terminal light absorber, transmitting energy exclusively to Nd^3+^ ions in the adjacent NP shell via Dexter transfer, followed by an energy migration step between shell Nd^3+^ ions and the nanocrystalline core. Partial energy localization in the core is presumed to result in the reversible energy transfer between Nd^3+^ ^4^*F*_3/2_ (~11, 460 cm^−1^) and Yb^3+^ ^2^*F*_5/2_ (10,400 cm^−1^), populating an equilibrium mixture of Yb^3+^ excited states under steady-state illumination; radiative deactivation of these states produces an NIR luminescence centered around 979 nm. Inspection of control data bolsters this narrative: NPs featuring a Nd^3+^/Yb^3+^-doped core and an undoped shell (NaGd_1−x−y_Nd_x_Yb_y_F_4_ | NaGdF_4_ -**343**) display no NIR emission (Figure 2B, inset). Yb^3+^-free controls (NaGd_1−x_Nd_x_F_4_ | NaGd_1−x_Nd_x_F_4_ -**343**) yield the intuitive result of an IR emission defined solely by Nd^3+^ transitions. UV illumination in the absence of the **343** ligand yields no detectable luminescence under identical conditions from either Nd^3+^ or Yb^3+^ lumiphores. Furthermore, ligand incorporation is shown to significantly increase luminescence yield upon excitation at 350 nm, with a resulting signal intensity at 979 nm approximately 1100-fold greater for the **343**-decorated particles than observed for the unmodified nanostructures (Figure 3). It is noteworthy that none of the higher energy emissions that typically result from transitioning between Nd^3+^ stark levels are found in the visible range between 360 and 860 nm (Koningstein and Geusic, 1964; Zhang et al., 2007, 2015; Chen et al., 2012; Li X. et al., 2013; Wang et al., 2015), a surprising fact, given the high density of energetic states in Nd^3+^ electronic structure, and a standing precedent in the literature of Nd^3+^-doped, rare-earth hosts yielding emission spectra reflective of the ion's several *f-f* transitions (Li X. et al., 2013; Mimun et al., 2013; Wang et al., 2015). One, or a combination, of four likely possibilities may explain such phenomena: (1) the overall equilibrium between Yb^3+^ ^2^*F*_5/2_ and the Nd^3+^ states serving as acceptor states from the **343** triplet manifold favors production of Yb^3+^ excited states; (2) The resonant exchange between Yb^3+^ ^2^*F*^5/2^ and Nd^3+^ donor states largely favors Yb^3+^ ^2^*F*_5/2_ production; (3) Nd^3+^ states are rapidly quenched by phonon-coupled effects involving either ligand or host lattice vibrational states; (4) Rates of energy migration between Nd^3+^ ions significantly outpace the rates of radiative decay from individual Nd^3+^ stark levels. The scope of data acquired in this current study make assertions (1) and (4) difficult to confirm, as it demands a comprehensive knowledge of the rates and efficiency of all steps from the initial point of photon absorption by the ligand to Yb^3+^ ion emission. However, the nature of energy exchange between Nd^3+^ and Yb^3+^ is moderately exothermic, with an energy gap that is sufficiently large to possibly make phonon-assisted, energetic back-transfer from Yb^3+^ (^2^*F*_5/2_) → Nd^3+^ (^4^*F*_3/2_) kinetically prohibitive in NaGd(Y)F_4_ hosts (1060 cm^−1^ energy gap vs. ~ 400 cm^−1^ phonon energy), suggesting (2) as a partial explanation. Finally, (3) also seems possible. Despite numerous reports of Nd^3+^ visible-frequency luminescence in rare-earth fluoride particles featuring surface ligands bound via Ln coordination by oxygen donors (Li X. et al., 2013; Wang et al., 2015), such studies are generally conducted in nonpolar solvents. In this work, the ethanol solvent needed for stabilizing particles with **343** contain OH oscillators that are likely conduits for non-radiative decays that may result in quenched visible emission form Nd^3+^. Such a possibility is also consistent with previous findings that the **343**-Nd^3+^ molecular complex dissolved in aqueous buffer only yields NIR Nd^3+^ luminescence (Sturzbecher-Hoehne et al., 2011).

### Excited State Dynamics and Energy Transfer

Extraction of **343**-Nd^3+^, Nd^3+^-Yb^3+^ energy transfer rates and Nd^3+^/Yb^3+^ excited state lifetimes were achieved through time-resolved luminescence measurements. Rates of energy transfer between the ligand and shell-confined Nd^3+^ were found through cryogenic (77 K) monitoring of the **343** triplet luminescence at 525 nm (Supplementary Figure 6). Mean decay times for the ligand triplet state in the presence of Nd^3+^ quenching were calculated according to Equation (**1**) (where I(t) is the time-dependent emission intensity, and I_0_ is its initial value), yielding values of 626 ± 48 μs. **343** triplet deactivation displayed tri-exponential behavior, occurring in decay phases with rates of 2197 ± 392 s^−1^, 382 ± 42 s^−1^, and 25.5 ± 19 s^−1^ at respective amplitudes of 0.60, 0.34, and 0.06, consistent with data previously reported for a **343**-sensitized, Eu^3+^-doped system (Agbo and Abergel, 2016; Agbo et al., 2016). Extracting mean lifetimes by calculating the weighted sum of these decay rates (Equation **2**) according to their respective contributions toward the measured decay spectrum yields a similar lifetime of 694 ± 127 μs, a value within error of the integral method. Ligand-Nd^3+^ energy transfer efficiencies were then determined according to Equation (**3**), where τ_DA_ is the measured **343** donor lifetime in the presence of Nd^3+^ acceptor ions and τ_D_ is the measured **343** donor lifetime in the absence of acceptor quenching. Values for τ_D_ were determined previously (1.61 ms) (Agbo and Abergel, 2016; Agbo et al., 2016). Accordingly, energy transfer between ligands on the NP surface and Nd^3+^ atoms in the nanocrystalline shell was found to proceed with an efficiency of 61 ± 3%.

(1) $$\begin{array}{l} <\tau >=\frac{1}{I_{0}}\int_{I_{0}}^{\;}I\left(t\right)dt \end{array}$$

(2) $$\begin{array}{l} <\tau >=\frac{1}{\sum_{j}k_{j}c_{j}} \end{array}$$

(3) $$\begin{array}{l} <\eta >=1-\frac{\tau_{DA}}{\tau_{D}} \end{array}$$

**Figure 4:**
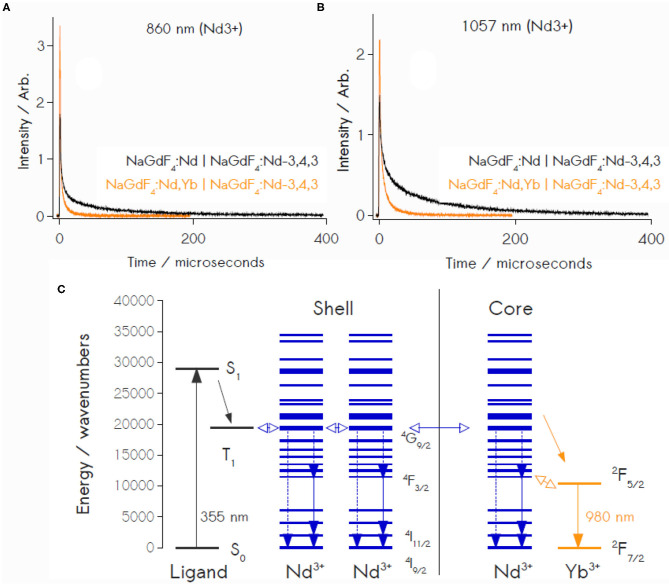
Time resolved Nd^3+^ luminescence at both 860 nm **(A)** and 1057 nm **(B)** reveals quenching in the presence of Yb^3+^ acceptors, confirming significant energy transfer between Nd^3+^ and Yb^3+^ ions. **(C)** Schematic Jablonski depiction of energy transfer processes in the sensitized Nd^3+^, Yb^3+^ core-shell particles. Downconversion involves direct transitioning from the ^4^*G*_9/2_ donor level in Nd^3+^ to the ^2^*F*_5/2_ level in Yb^3+^.

Quenching of Nd^3+^ excited states by Yb^3+^ ions was resolved through monitoring of Nd^3+^ transitions at 860 nm (^4^*F*_3/2_ → ^4^*I*_9/2_) and 1057 nm (^4^*F*_3/2_ → ^4^*I*_11/2_), as the ^4^*F*_3/2_ state in Nd^3+^ lies at a similar energy to the ^2^*F*_5/2_ state of Yb^3+^ (Figure 4C). Transient luminescence of the 860 nm transition proceeds in two phases, with treatments showing rates of 2.13 x 10^5^ ± 6150 s^−1^ and 798 ± 67.7 s^−1^; ^4^*F*_3/2_ → ^4^*I*_11/2_ decay at 1057 nm displays quenching rates of 1.56 x 10^5^ ± 2760 s^−1^ and 4940 ± 140 s^−1^ (Figures 4A,B). Controls containing no core Yb^3+^ (NaGdF_4_:Nd|NaGdF_4_:Nd-**343** core|shell NPs) yielded Nd^3+^ decay rates of 4.14 x 10^5^ s^−1^ at 860 nm and 1.43 x 10^5^ s^−1^ at 1057 nm. These data permit the calculation of Nd^3+^-Yb^3+^ energy transfer efficiencies (Equation **3**) of 86.8 ± 2.0 and 82.4 ± 1.4%, using the respective 860 nm and 1057 nm transitions as spectroscopic handles (Supplementary Figure 7).

### Downconversion Order *via* Power Dependence

**Figure 5:**
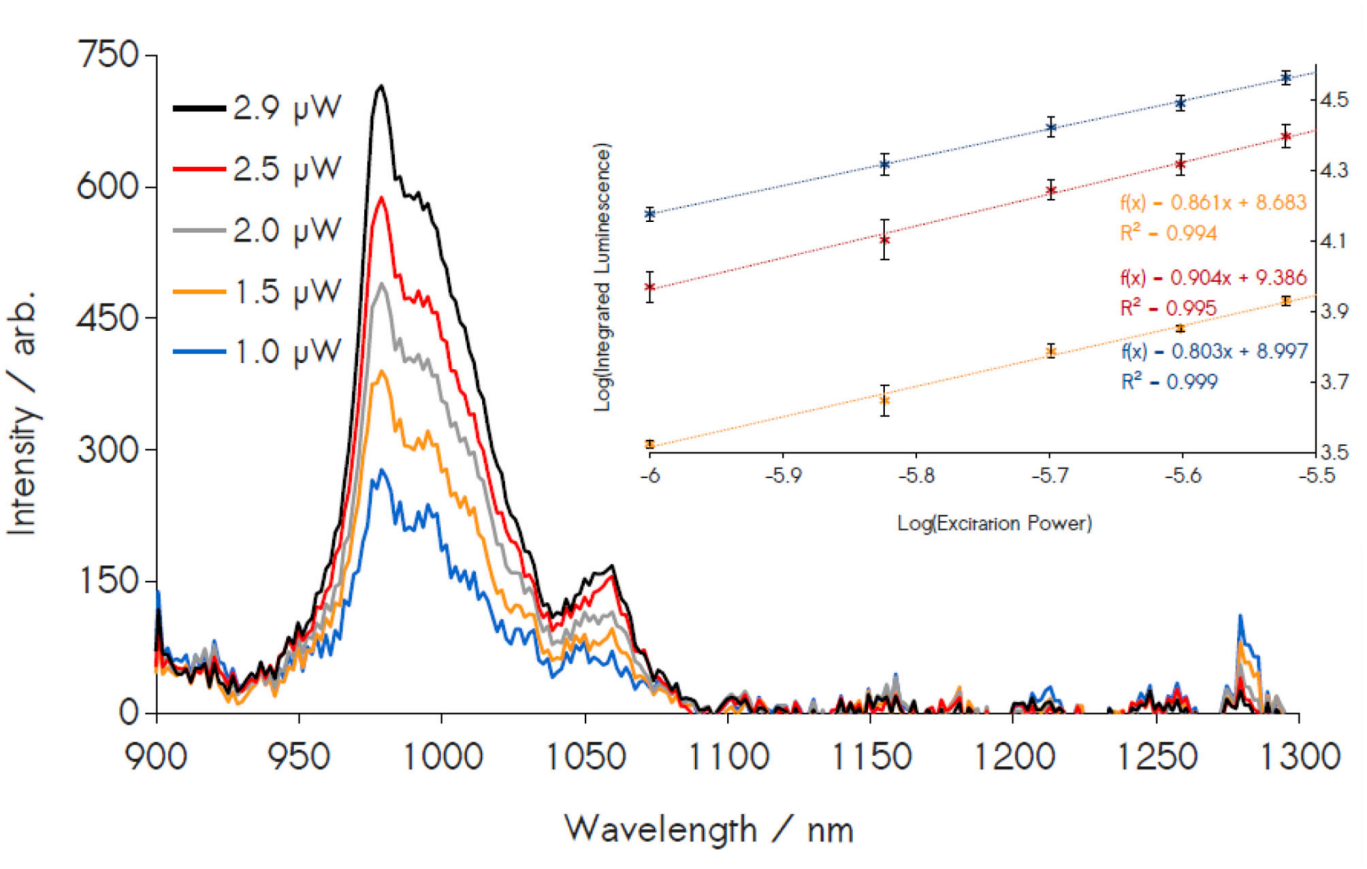
Power dependence study of 3/20% doped Nd^3+^/Yb^3+^ NPs. Emission spectra are integrated in the region of Yb^3+^ luminescence (940–1040 nm) and plotted against the excitation power (inset). The power dependence of these material exhibits a sub-linear slope, with a total of 10 independent measurements yielding an average slope of 0.86 ± 0.04 among three replicates, confirming the presence of two-photon emissions.

Luminescence measurements as a function of incident light power were conducted to determine the order of photon production by the coated nanocrystals featuring Nd^3+^ and Yb^3+^ doping levels of 3 and 20%, respectively (Figure 5) (Suyver et al., 2005; Zou et al., 2012; Lin et al., 2015). Luminescence measured at light intensities over the range of 6.2–2.1 mW cm^−2^ (3.0 μW to 1.0 μW as measured at the fiber optic) displays linear correlations between the source power logarithms and integrated emission intensity in the NIR regime, with an average slope of 0.86 ± 0.04 (Supplementary Figures 8–9). Notably, this behavior occurs under diffuse illumination conditions relevant to the terrestrial solar spectrum (~100 mW cm^−2^ total), a stark contrast to the high excitation powers generally required for Ln luminescence. Furthermore, the excitation powers used here are far below the regimes for power saturation that are generally observed in these and related materials (Suyver et al., 2005; Chen G, et al., 2009; Lin et al., 2015; Loiko et al., 2016; Chen et al., 2017), demonstrating that our reported power dependencies are not mere artifacts of excited state saturation. The observed sub-unity slopes are diagnostic of a multi-photon production process involving direct transitions from the ^4^*G*_9/2_ donor level in Nd^3+^ to the ^2^*F*_5/2_ level in Yb^3+^. Generally, an idealized two-photon DC process yields a theoretical slope of *n* = 0.5, in contrast to the slope of 1 found in the power dependence for a typical, 1:1 Stokes-shifted emission and the *n* = 2 slope for two-photon UC (Suyver et al., 2005; Chen G, et al., 2009; Zou et al., 2012; Lin et al., 2015; Loiko et al., 2016; Chen et al., 2017). Here, the intermediate slope observed in the NIR luminescence suggests a photoemission that is a function of both 1:1 Stokes-shifted luminescence and 1:2 photon DC pathways. Furthermore, while our slopes values are far from the ideal two-photon slope, the small measurement error strongly suggests the measured effect is a statistically significant one, and that the multi-photon effect is therefore genuine (if minor); this is bolstered by control power dependencies conducted with Nd^3+^-only, **343** NPs, which yield a slope value of 1, for Nd^3+^ luminescence detected at 1057 nm (Supplementary Figure 10). The relative contributions of these various decay channels to the overall photoluminescence are difficult to precisely quantify but are estimated with the system described in Equation (4), where terms a_k_ are weights representing the respective contributions of the k^th^ unique decay channel (i.e., 1:1 photon emission, 2-photon DC), m_k_ are the theoretical slopes for a photon DC process of order 1/m_k_ and m is the total slope derived from power dependence, a value in which the contributions of all active luminescent decay channels are implicit. For our particular case, where only two unique processes (one-photon, m = 1 luminescence and m = 0.5, two-photon conversion) are present, solving for the coefficients for m = 0.86 yields a_1_ = 0.72 and a_2_ = 0.28, suggesting that, while two-photon mechanisms are operative (28%), luminescence in the NIR remains dominated by single-photon processes (72%) (Strek et al., 2001). These results are notable, as a handful of previous reports had pointed to claims of ligand-sensitized, Stokes-shifted luminescence in nanocrystals, however these studies had not produced any explicit evidence of multi-photon DC (Charbonnière et al., 2008; Cross et al., 2010; Chen et al., 2013; Li S. et al., 2013, 2014; Irfanullah et al., 2015; Goetz et al., 2016).

(4) $$\begin{array}{l} m=\underset{k}{\sum }a_{k}m_{k};1=\underset{k}{\sum }a_{k} \end{array}$$

### Quantum Efficiency of Sensitized Emission

**343**'s efficacy as a photosensitizer was further evaluated by determining the internal quantum yields observed for Yb^3+^ infrared emission. Direct Nd^3+^ excitation was achieved through laser excitation at 456 nm ((^4^*I*_9/2_ → ^4^*G*_9/2_, SI). Direct excitation pathways display the low external quantum yields that are a natural consequence of the low *f-f* absorptivities of Ln transitions, with direct generation of Nd^3+^ excited states yielding an average efficiency of 0.15, 0.14, and 0.34% (0.21 ± 0.11%) for Yb^3+^ luminescence integrated over the range 940–1040 nm. Using the relation Φ_343−*Yb*_ = Φ_*Nd*−*Yb*_η_*sens*_, where η_*sens*_ is the ligand-Nd^3+^ sensitization efficiency, allows for determination of an approximate quantum yield of 0.13% for Yb^3+^ emission originating from 355 nm ligand excitation. In addition to our measured quantum yields, we compare these results to a treatment for calculating DC quantum yields commonly employed throughout photon DC literature (Vergeer et al., 2005; Lakshminarayana et al., 2008a,b; Ye et al., 2008, 2011; Chen X, et al., 2009; Van Der Ende et al., 2009; Li K.-Y. et al., 2014; Zhu et al., 2014). As defined in Equation (**5**), *QY*_Nd−Yb_ is the quantum efficiency of DC from a Nd^3+^ donor to an Yb^3+^ acceptor, whereas η_*Nd*_ is the Nd^3+^ donor quantum efficiency and is generally assumed to be 100% (no non-radiative losses); (Vergeer et al., 2005; Lakshminarayana et al., 2008a,b; Ye et al., 2008, 2011; Chen X, et al., 2009; Van Der Ende et al., 2009; Li K.-Y. et al., 2014; Zhu et al., 2014). *ETE* is the Nd^3+^-Yb^3+^ energy transfer efficiency (0.87). Here, such approach yields a maximum two-photon quantum yield of 182% for these nanocrystals in the case of luminescence driven by photoexcitation of the Nd^3+^ absorption band at 456 nm. Determination of the ligand-sensitized Yb^3+^ quantum yields are found by factoring in the additional ligand-Nd^3+^ energy transfer step according to Equation (6). Applying our experimentally determined value of 61% for the ligand-Nd^3+^ sensitization efficiency yields a maximum quantum yield of 114% for a two-photon Yb^3+^ emission in these crystals, where the ligand is the terminal light absorber, rather than Nd^3+^. It must be stressed that such values of two-photon emission that are calculated using Equations (**5**, **6**) only represent theoretical upper limits on the two-photon quantum yields, as they assume the absence of non-radiative decay paths (Vergeer et al., 2005; Lakshminarayana et al., 2008a,b; Ye et al., 2008, 2011; Chen X, et al., 2009; Van Der Ende et al., 2009; Li K.-Y. et al., 2014; Zhu et al., 2014). Here, we have provided the actual quantum yields of these materials as a comparison, with the substantial gap between the calculated and experimental values reflecting the considerable degree of non-radiative losses that exist in these nanocrystals. This observation should prompt a conservative interpretation of any two-photon quantum efficiencies determined through calculated methods assuming an absence of non-radiative decays. Such approaches run in stark contrast to physical measurements of two-photon production, where an accounting of non-radiative excited-state deactivation is necessarily an implicit feature of acquired experimental data.

(5) $$\begin{array}{l} QY_{Nd-Yb}=\eta_{Nd}\left(1-ETE\right)+2\left(ETE\right) \end{array}$$

(6) $$\begin{array}{l} QY_{343-Yb}=\eta_{sens}\times QY_{Nd-Yb} \end{array}$$

## Conclusion

This work demonstrates the value in considering not only the respective energies of Ln donor/acceptor levels in the design of multi-photon emitters, but also the role that core-shell structures can play. Specifically, the use of layered crystal domains permits the critical segregation of ligand absorber and metal donors/acceptors (Nd^3+^/Yb^3+^), enabling an efficient, stepwise ligand-donor-acceptor energy transfer that mitigates the possibility of quantum efficiency losses arising from the direct sensitization of Yb^3+^ by **343** (Sturzbecher-Hoehne et al., 2011). Though availability of a resonant ^4^*F*_3/2_ donor level in Nd^3+^ facilitates energy transfer to the Yb^3+^ excited state, the high density of Nd^3+^ *f*-states falling between energies of the **343** triplet (21,500 cm^−1^) and the Nd^3+4^ *F*_3/2_ donor (11,460 cm^−1^) allows for significant losses through multi-phonon relaxation during **343** → Nd^3+^ sensitization, a physical basis for the low contribution of two-photon emission to the overall Yb^3+^ luminescence (while the measured energy transfer efficiency for this step is 0.61, this is only a remark on the total energy siphoned from **343** phosphors by Nd^3+^ acceptor levels; it provides no information on non-radiative losses incurred by excited Nd^3+^ ions during internal conversion to yield the ^4^*F*_3/2_ intermediate). These results build on our initial studies of ligand sensitization of Eu^3+^ in NaGd(Y)F_4_ crystals (Agbo et al., 2016), extending the general principle of sensitized Ln NP luminescence to the particular case of two-photon generation in the NIR. The present findings detail a material capable of shifting luminescence over a range of 600 nm, from UV to IR, with a NIR emission profile that is a partial function of two-photon processes. These results bear significance to the challenge of addressing energetic mismatches between terrestrial solar illumination and the spectral response of commercial photovoltaics.

## Data Availability Statement

All datasets generated for this study are included in the article/ Supplementary Material.

## Author Contributions

PA and RA designed the research. PA performed experimental work. All authors performed data analysis and contributed to the writing of the manuscript.

## Conflict of Interest

RA and PA are listed as inventors on a patent application filed by the Lawrence Berkeley National Laboratory and describing inventions related to the research results presented here. The remaining author declares that the research was conducted in the absence of any commercial or financial relationships that could be construed as a potential conflict of interest.

## Supplementary Material

**Supporting Information**: Experimental procedures, quantum yield data, time-resolved luminescence, energy transfer calculations, and Eu^3+^-Cm^3+^ excitation spectra are included in the Supporting Information. This material is available free of charge via the internet at http://pubs.acs.org.
