# Supplementary material for: Infrared Photon Pair-Production in Ligand-Sensitized Lanthanide Nanocrystals
Source: Front Chem. 2020 Nov 4;8:579942. doi: 10.3389/fchem.2020.579942 (PMC7672211; doi:10.3389/fchem.2020.579942)
Supplement: Supplementary file 1 [file Data_Sheet_1.docx]

Infrared Photon Pair-Production in Ligand-Sensitized Lanthanide Nanocrystals

Peter Agbo, ^†^ Jacob S. Kanady, ^‡^ and Rebecca J. Abergel*^,†,┴^

^†^Chemical Sciences Division Lawrence Berkeley National Laboratory, Berkeley CA 94720

^‡^Department of Chemistry, University of California, Berkeley, CA 94720

^┴^Department of Nuclear Engineering, University of California, Berkeley, CA 94720

AUTHOR INFORMATION

Corresponding Author

*abergel@berkeley.edu

**Table of Contents**

Experimental Procedures 03

Results and Discussion 05

Nanoparticle Characterization - TEM 05

Nanoparticle Characterization - Powder X-ray Diffraction 09

Time-Resolved Ligand Luminescence 10

Nd^3+^, Yb^3+^ Time-Resolved Luminescence 13

Spectral Power Dependence 18

Power Measurement Spectra 21

Power Density Determination 26

References 27

Author Contributions 27

**Experimental Procedures**

*Nanoparticle Synthesis and Ligand Modification:* Nanoparticles were synthesized as described in the protocols of Wang *et al*.^1^ Briefly, nanoparticle core structures were synthesized first, with typical starting mixtures of 3.08 ml Gd(CH_3_CO_2_)_3_•xH_2_O (Sigma-Aldrich), 800 μl Yb(CH_3_CO_2_)_3_•xH_2_O (Sigma-Aldrich) and 120 μl of Nd(CH_3_CO_2_)_3_•xH_2_O in 8 ml 1-oleic acid (Alfa Aesar) and 12 ml 1-octadecene (90%, Sigma-Aldrich). Nanoparticle aliquots (2 ml) in cyclohexane were precipitated by the addition of 2 ml of ethanol. Nanoparticle pellets were collected by centrifugation at 13,000 rpm for 5 min. Following solvent removal, the pellets were resuspended in 2 ml ethanol using sonication. Centrifugation was then repeated and the solvent decanted. Fresh ethanol (2 ml) was used to resuspend the particles before their addition to a round bottom flask. Afterwards, 1 ml of 7.5 mM 3,4,3-LI(1,2-HOPO) (Ash Stevens, Inc.) in pH 6.0 50 mM Hepes buffer was added. The mixture was stirred overnight at room temperature to allow for ligand binding to the nanoparticles. Following ligand incubation, samples were washed at least four times in ethanol using a sequence of centrifugation and resuspension via sonication.

*Steady-State NIR Photoluminescence and Power Dependence:* Power dependence data were collected by illumination of nanoparticle samples with light from a Jobin Yvon Horiba Fluorolog 3 system equipped with a xenon arc lamp. The excitation wavelength set to 355 nm with a 14-nm bandpass. Infrared emission from the samples was detected using an Ocean Optics NIR detector connected to the Fluorolog sample chamber using a fiber optic positioned orthogonally with respect to the excitation beam. Detector settings were as follows: 30 s integration times averaged over 3 individual scans and a boxcar setting of 2. The detector’s thermoelectric peltier was set to -17°C to minimize thermal noise in the acquired data. A Thorlabs s120c power meter was connected to the Fluorolog sample chamber with a fiber optic sitting in the path of the excitation beam, after the sample. A rotary, continuously-variable neutral density filter was installed between the sample cuvette and the excitation source (in the same path as the power meter) to grant fine control over the total light intensity used to illuminate the sample. Rotation of the filter allowed reliable attenuation of the light over the range from 3.0 μW to 125 nW, as measured through the fiber optic (Ocean Optics, ~0.8 mm aperture). However, a lower limit of 1.0 μW was used to ensure a suitable signal to noise ratio in the collected spectra. Measurement of light intensity at the sample yielded a mean power density of 6.2 mW cm^-2^ (Figure **S11**). Power data were collected by averaging over the course of 1 min, with the meter’s detection wavelength set to 355 nm. Multi-photon emission from the sample was evidenced by constructing double-logarithmic plots of source excitation power versus integrated sample emission intensity. Spectral integrals were evaluated at wavelengths relevant to Yb^3+^ luminescence (900-1040 nm).

*Time-Resolved Photoluminescence:* Determination of ligand phosphorescence decay times in the presence of Nd^3+^ was measured on the Fluorolog 3 system in MCS lifetime mode. Samples were cooled to 77 K in a liquid nitrogen cryostat. Excitation parameters used were similar to those described previously: a 355-nm excitation signal with a 14-nm bandpass sourced from the Fluorolog's xenon arc lamp, coupled with a 1-nm emission bandpass centered at 525 nm. Emission was measured using a Horiba PPD850 photomultiplier tube (PMT). The time domain for measurements was set at 10 μs channel-1 and 3,000 channels sweep-1 (30.0 ms observation window). Time-resolved data were fit using exponential decay functions in MATLAB. Nd^3+^ and Yb^3+^ lifetimes were measured on an Edinburgh Instruments photospectrometer. Sample excitation was achieved using a tunable laser as a pump source (OPOtek). An upper wavelength limitation of the OPO precluded laser excitation of the ligand. As a result, metal luminescence lifetimes were found through direct f-f excitation of Nd^3+^ at 456 nm (transition) and 100% laser power. Typical instrument settings were as follows: 4 ms window, 2,000 channels, 0.3 ms delay, 10-nm excitation slits, using a NIR (ExtRed 750) PMT for monitoring Yb^3+^ emission at 980 nm; 100 μs, 2000 channels, 0.048 ms trigger delay, 10 nm slits (ExtRed 750 detector) for measuring Nd^3+^ emission at 860 nm. Nd^3+^ emission at 1057 nm was determined using the same instrument parameters as those for acquisitions at 860 nm except for the use of an LN_2_-cooled, NIR PMT (NIR1200).

*Quantum Yields:* External quantum yield measurements for both ligand and metal-centered excitation were performed with a custom-made spectrometer. For ligand-excited quantum yields, samples were illuminated with a 340-nm led (Thorlabs). Quantum yields were measured using direct Nd^3+^ excitation, with a Fianium multi-spectrum laser tuned to 456 nm. Measurement of the excitation beam was done using an Si photodiode with a detection wavelength centered at 540 nm, along with gain and binning factors set to 1. In both cases, NIR emission from Yb^3+^ and Nd^3+^ was detected using the Si photodiode centered at 900 nm. Data was collected using integration times of 300 s, a gain factor of 1 and binning factor of 400.

*Transmission Electron Microscopy:* TEM samples were prepped by drop-coating solutions of nanoparticles suspended in either ethanol (for ligand-containing samples) or cyclohexane (for unmodified nanoparticles) onto copper TEM grids, followed by drying in ambient atmosphere. Imaging analysis was conducted using an LaB6 Tecnai G2 20 transmission electron microscope operating at 200 kV.

*Powder X-ray Diffraction:* X-ray diffraction data were collected on a Rigaku diffractometer equipped with a Cu k-alpha x-ray source (54 angstrom wavelength) and a zero-background sample plate. Samples were prepped by drying the nanoparticle samples on a sand bath at 50^o^C and collecting the resulting powders. Bragg peaks were referenced versus ICSD archives of β-NaGdF_4_ diffraction datasets to confirm synthesis of the hexagonal crystal phase.

**Results and Discussion**

**Figure S1.** Nanoparticle TEM Characterization.


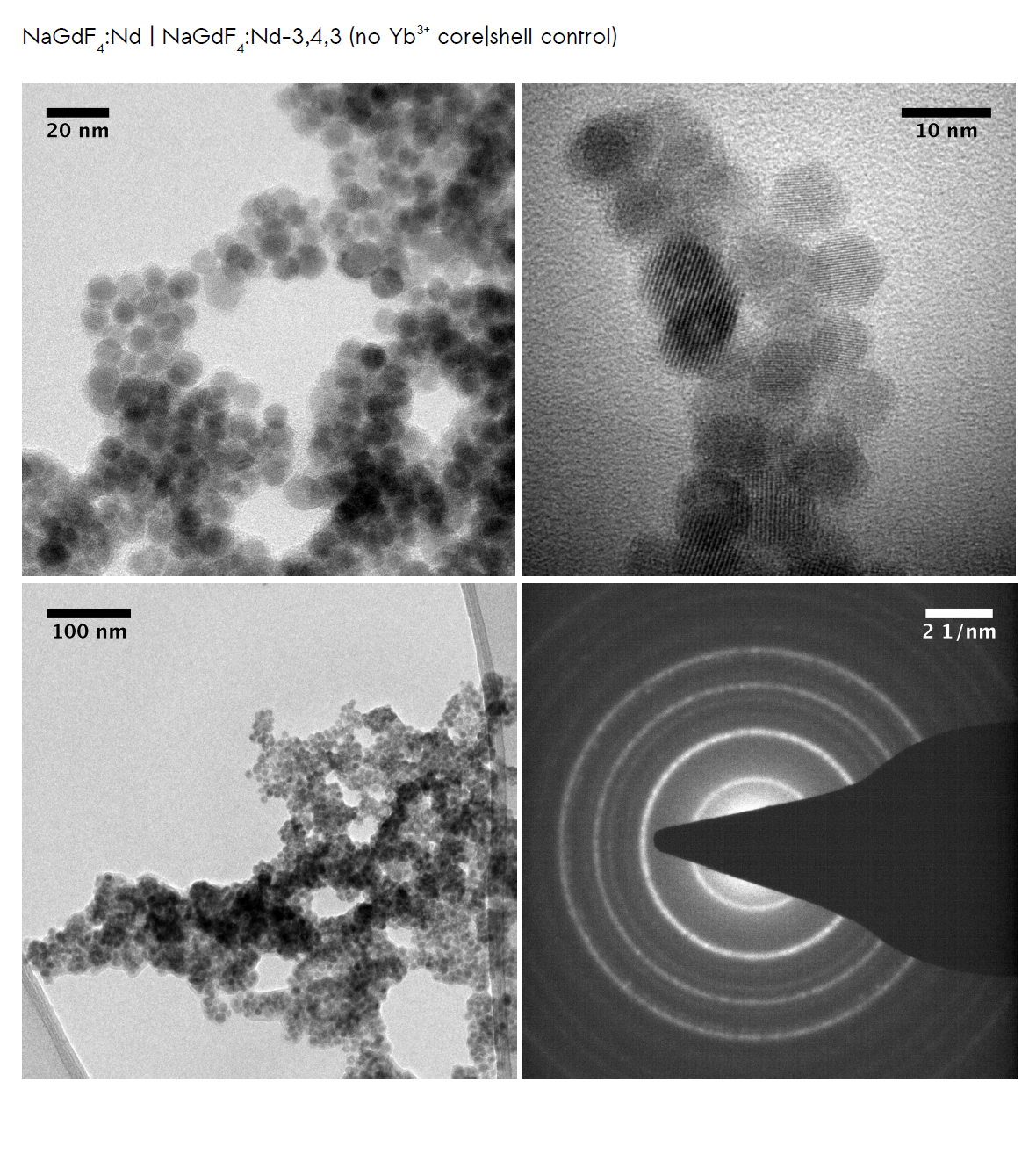


**Figure S2.** Nanoparticle TEM Characterization.


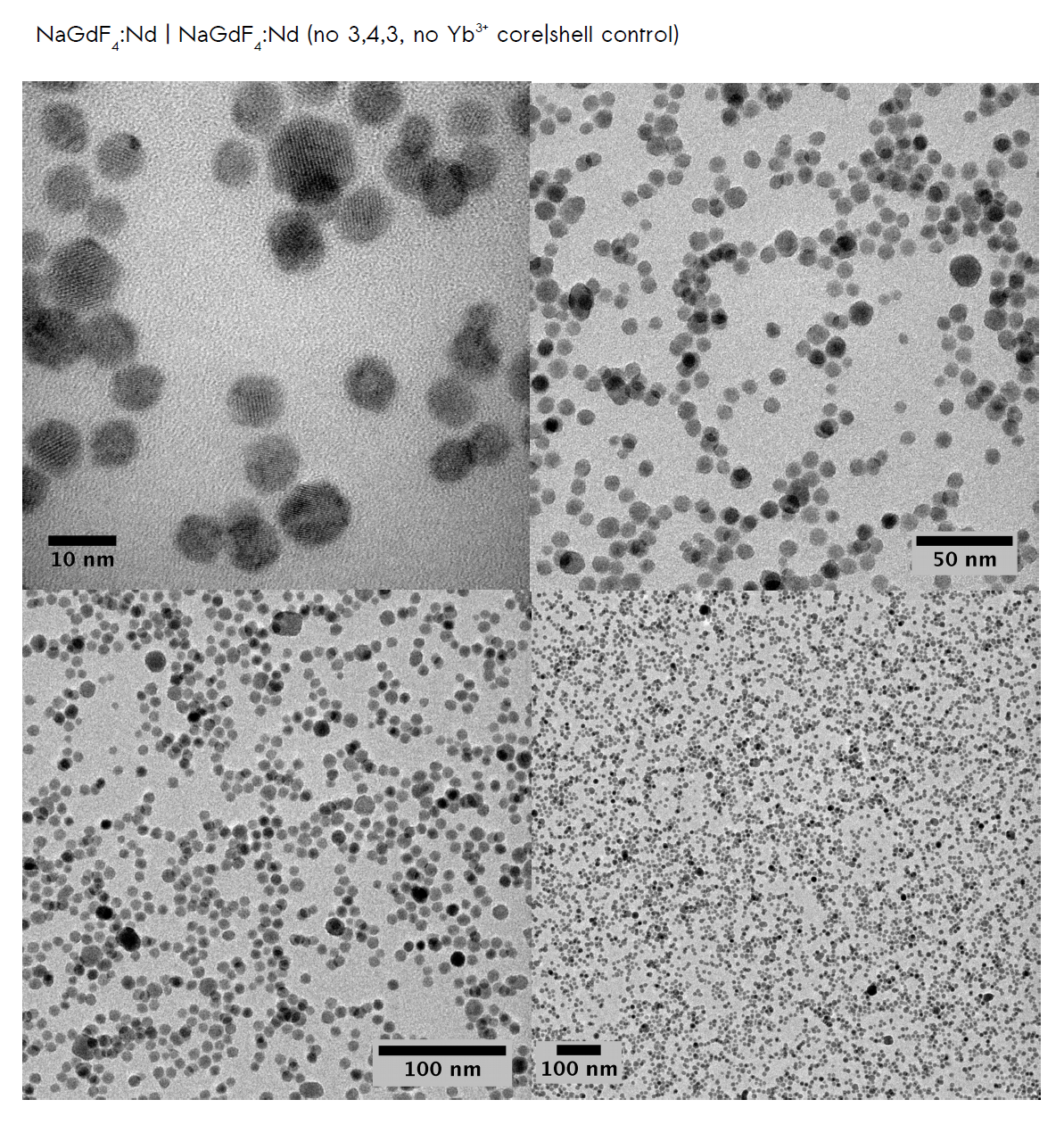


**Figure S3.** Nanoparticle TEM Characterization.


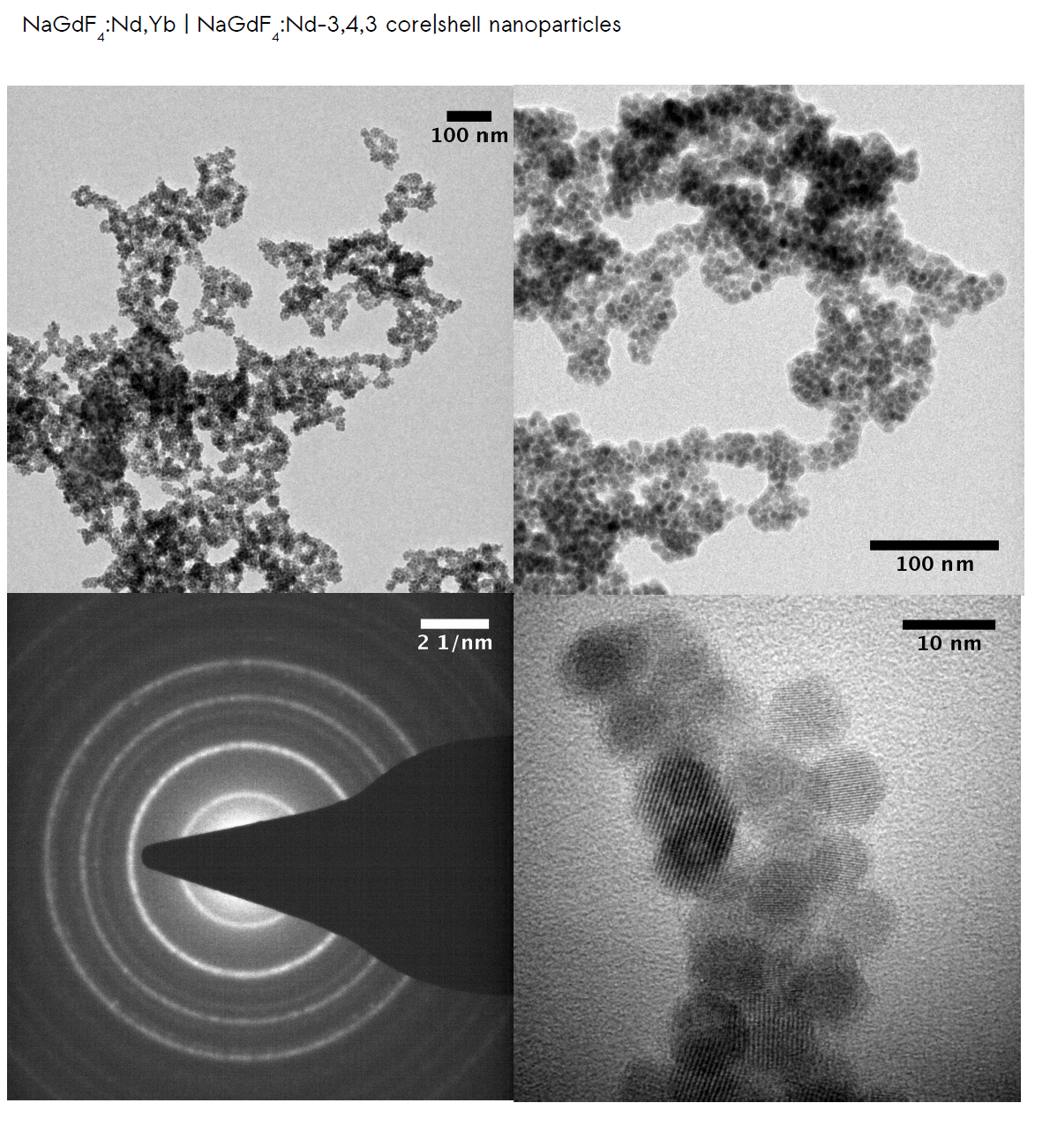


**Figure S4.** Nanoparticle TEM Characterization.


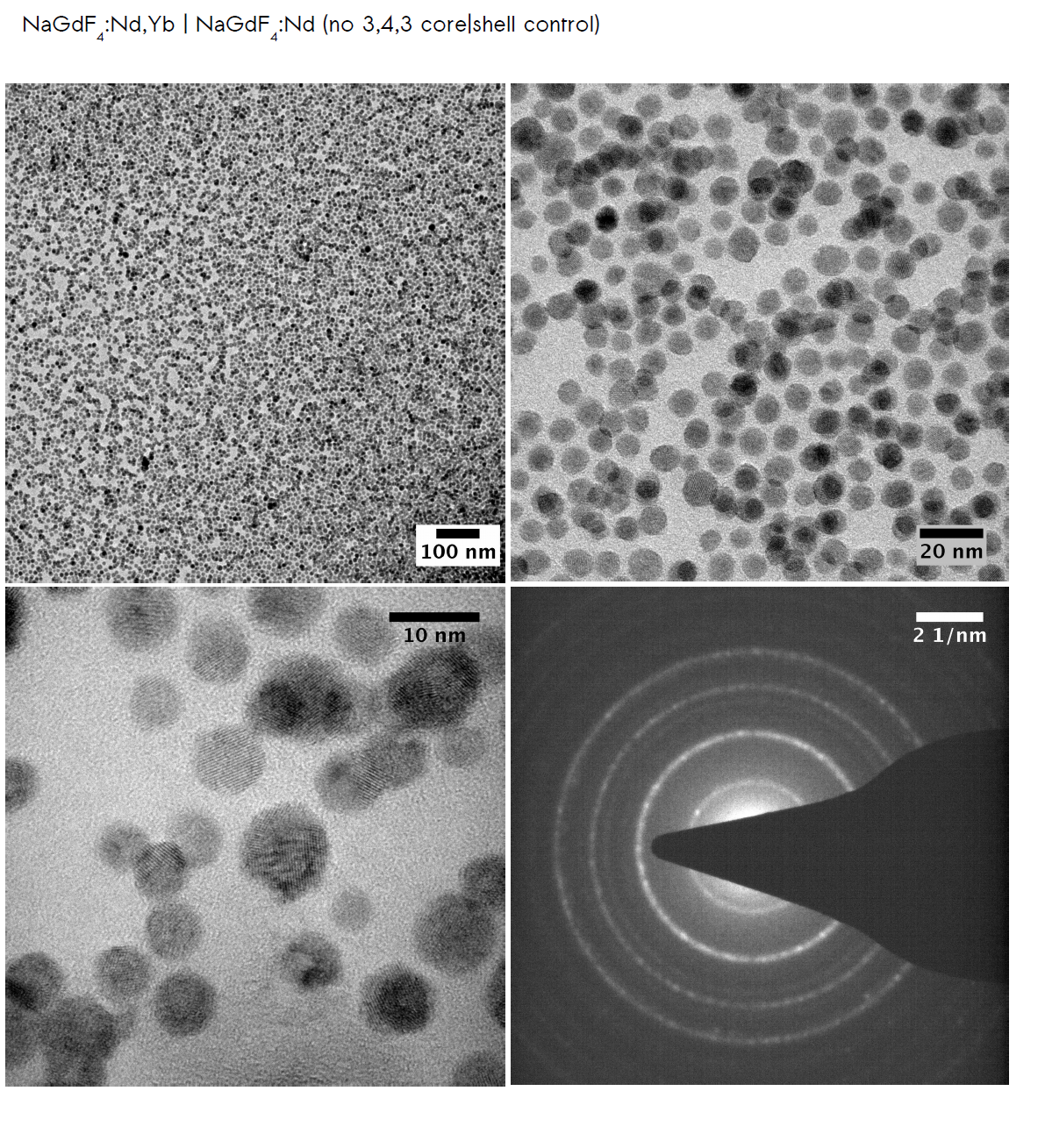


**Figure S5.** Nanoparticle Characterization – Powder X-ray Diffraction.


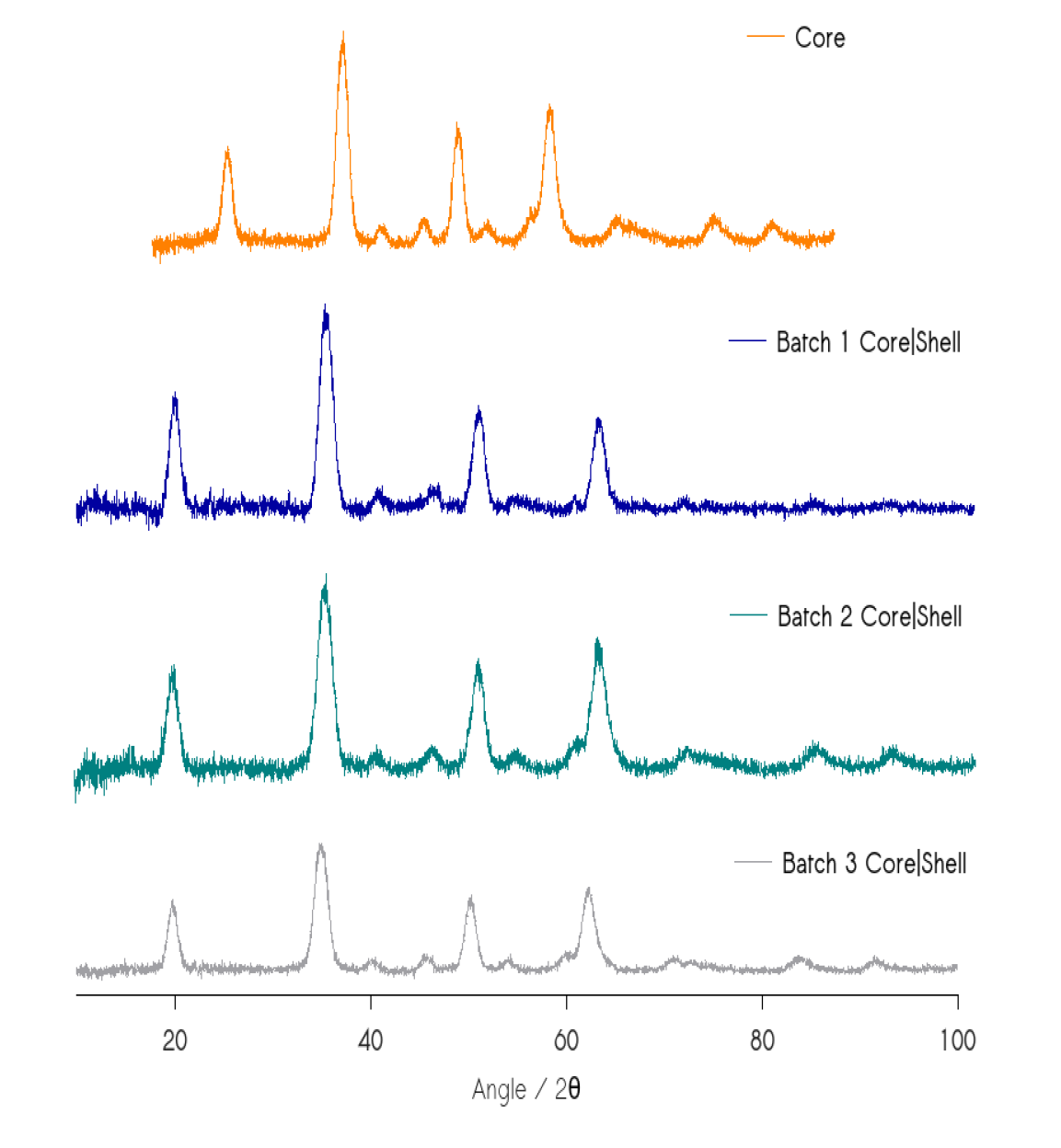


**Figure S6. Time-Resolved Ligand Luminescence.** 77K monitoring of 525-nm 3,4,3 triplet decay on NaGdF4:Nd,Yb | NaGdF4:Nd-3,4,3 core | shell nanoparticles, and corresponding fits for three independent batches (A, B, C) of nanparticles.

**A)**


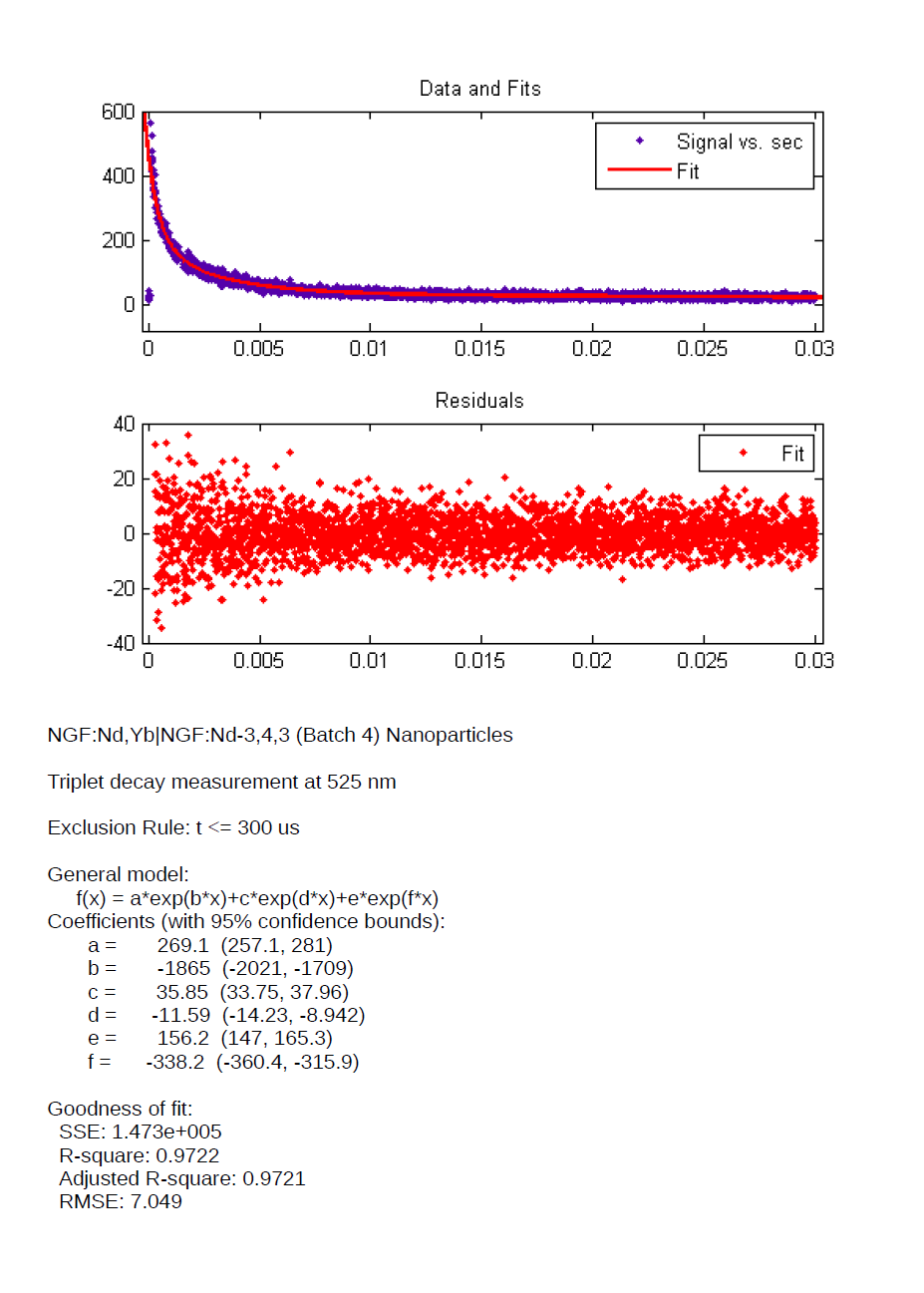


**B)**


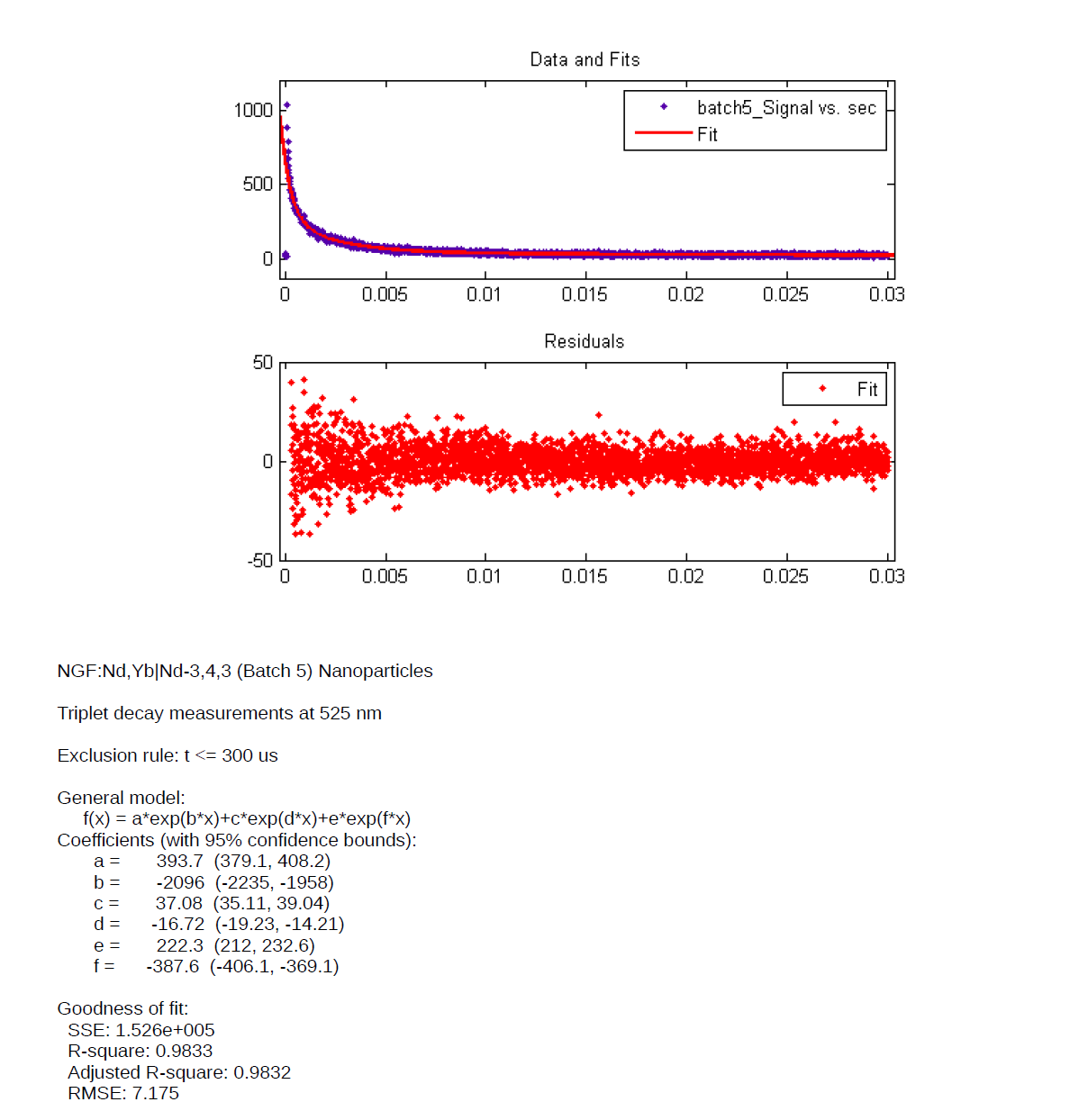


C)


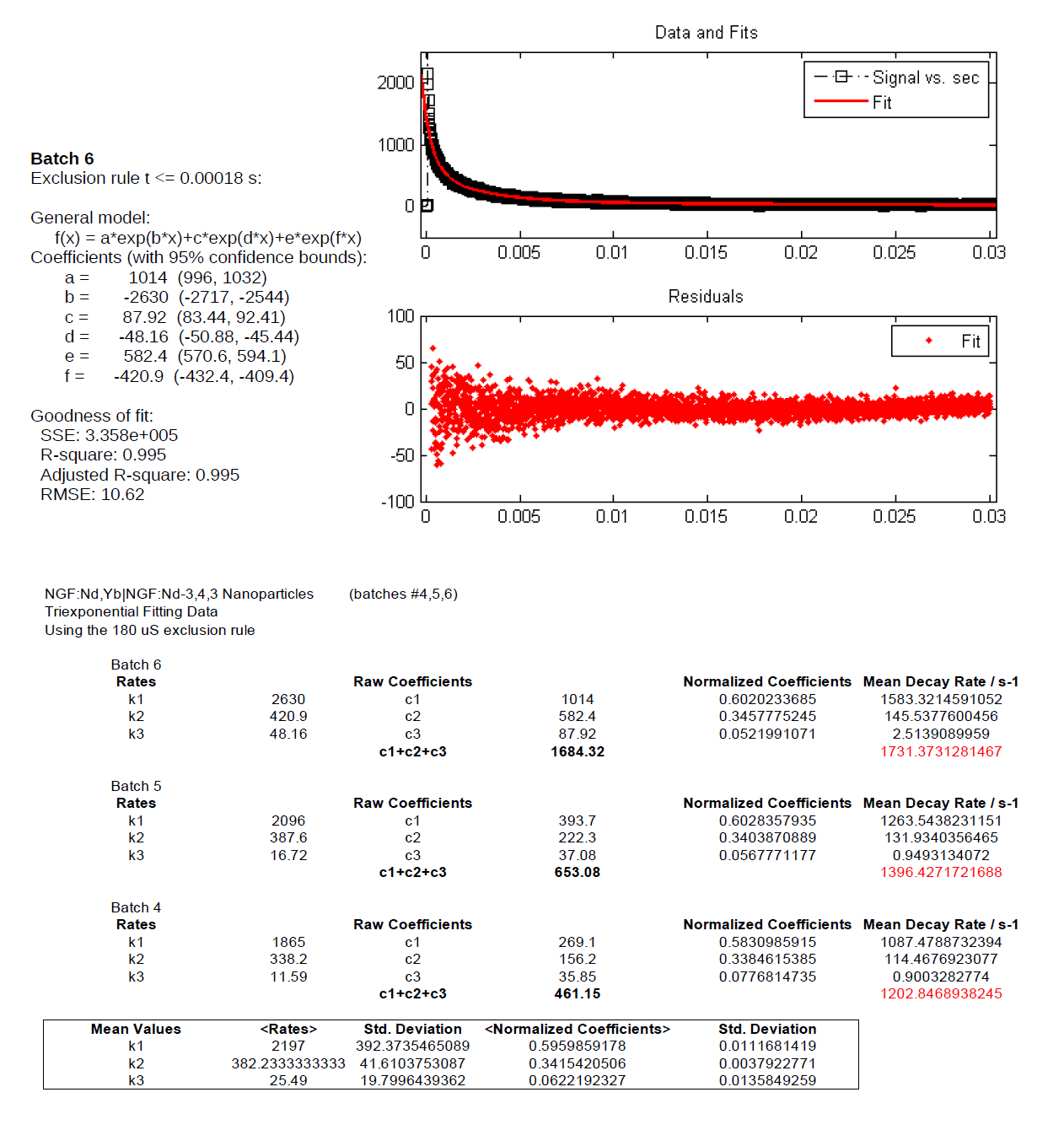


**Figure S7.** **Nd^3+^, Yb^3+^ Time-Resolved Luminescence.** Time-resolved, RT monitoring of Nd^3+^ luminescence quenching at 1057 nm and 860 nm on NaGdF_4_:Nd,Yb | NaGdF_4_:Nd-3,4,3 core | shell nanoparticles, referenced versus unquenched, no-acceptor (NaGdF_4_:Nd | NaGdF_4_:Nd-3,4,3) controls. Corresponding fits are detailed subsequently.


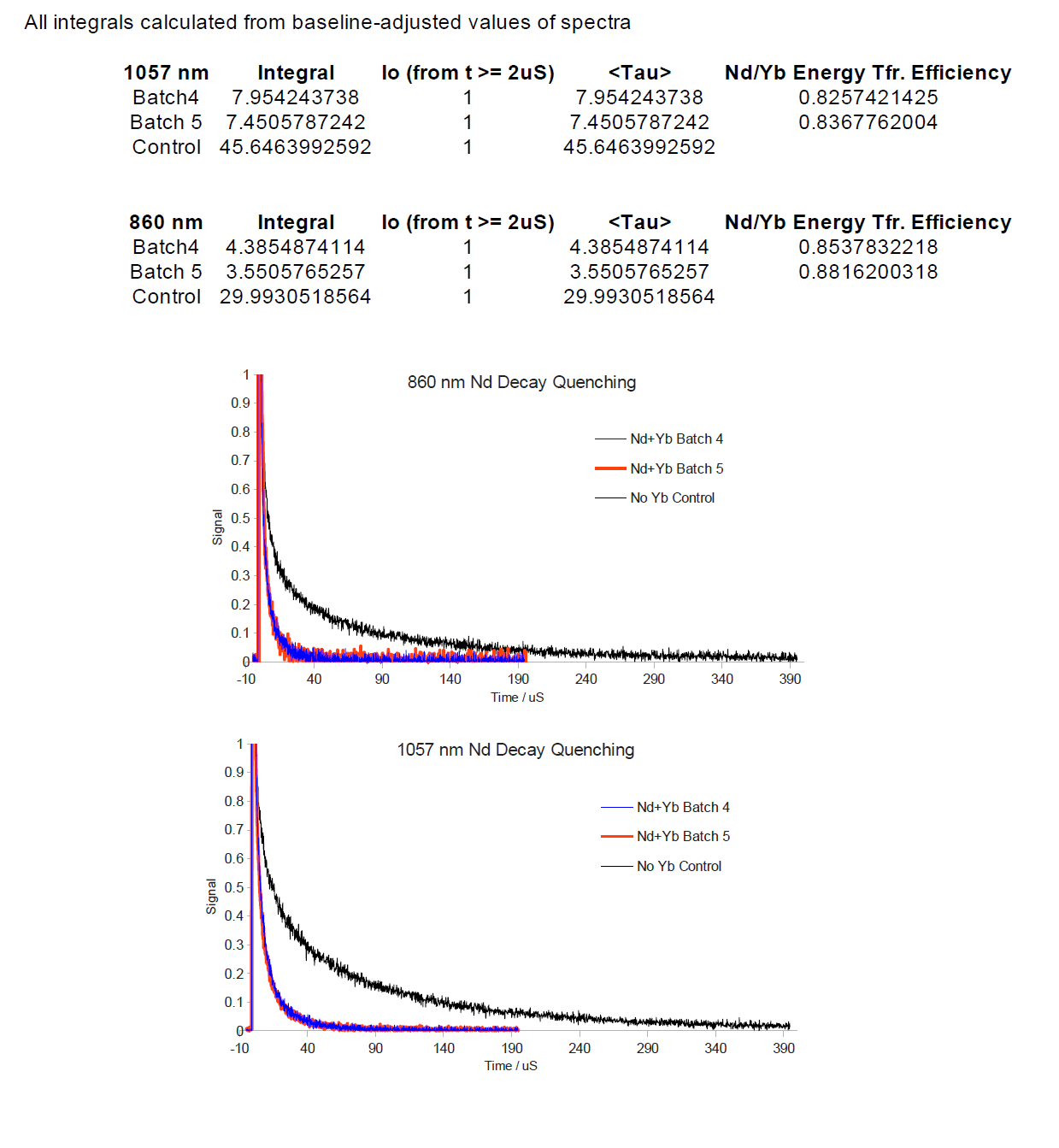


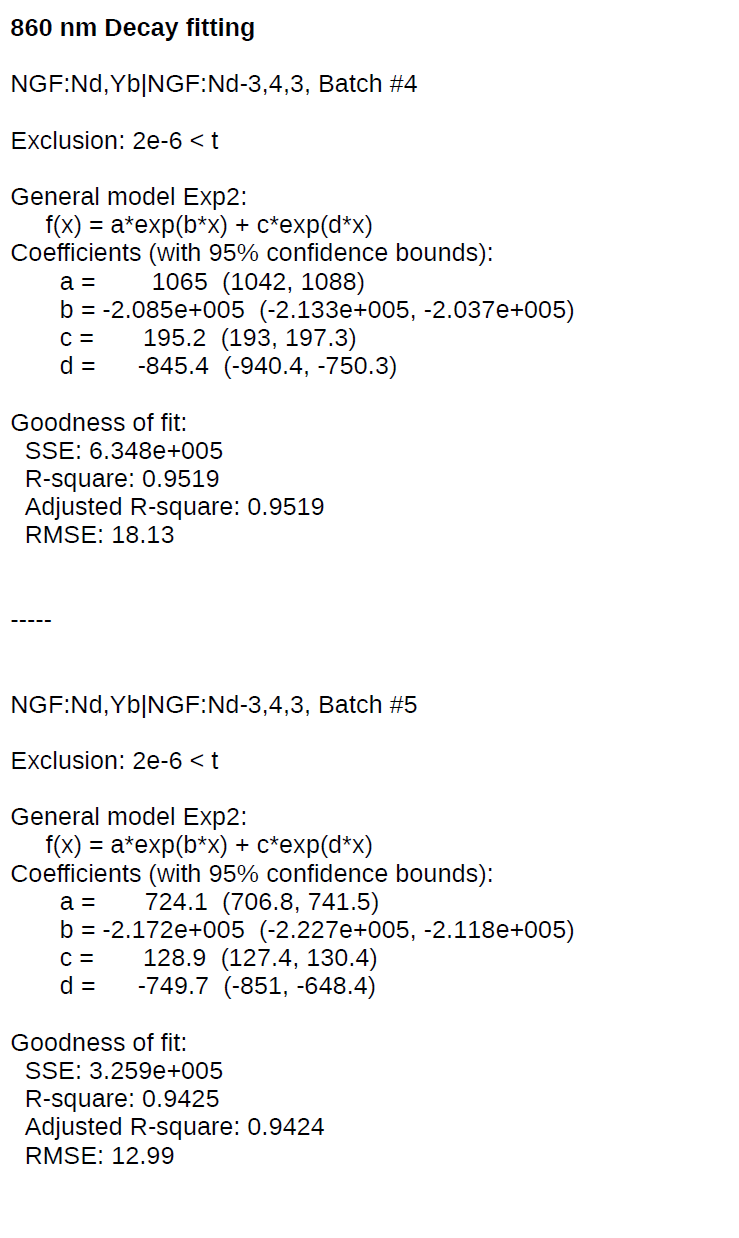

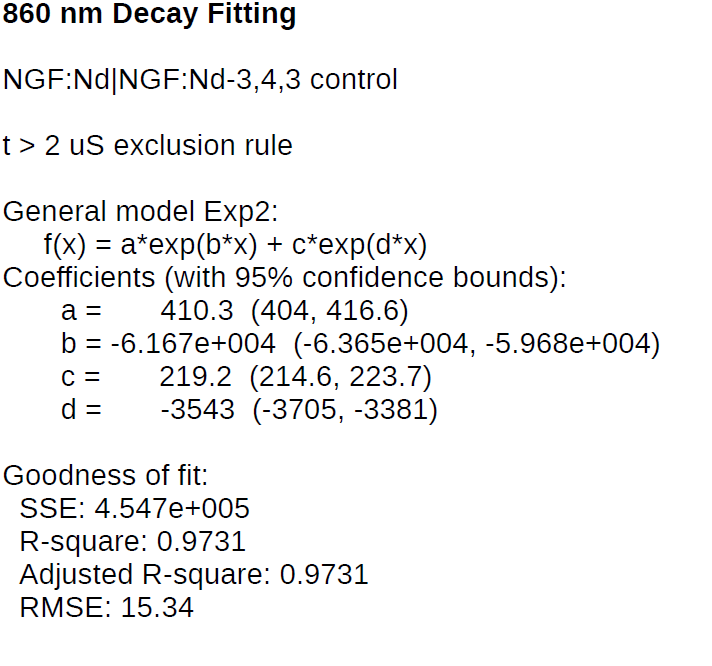

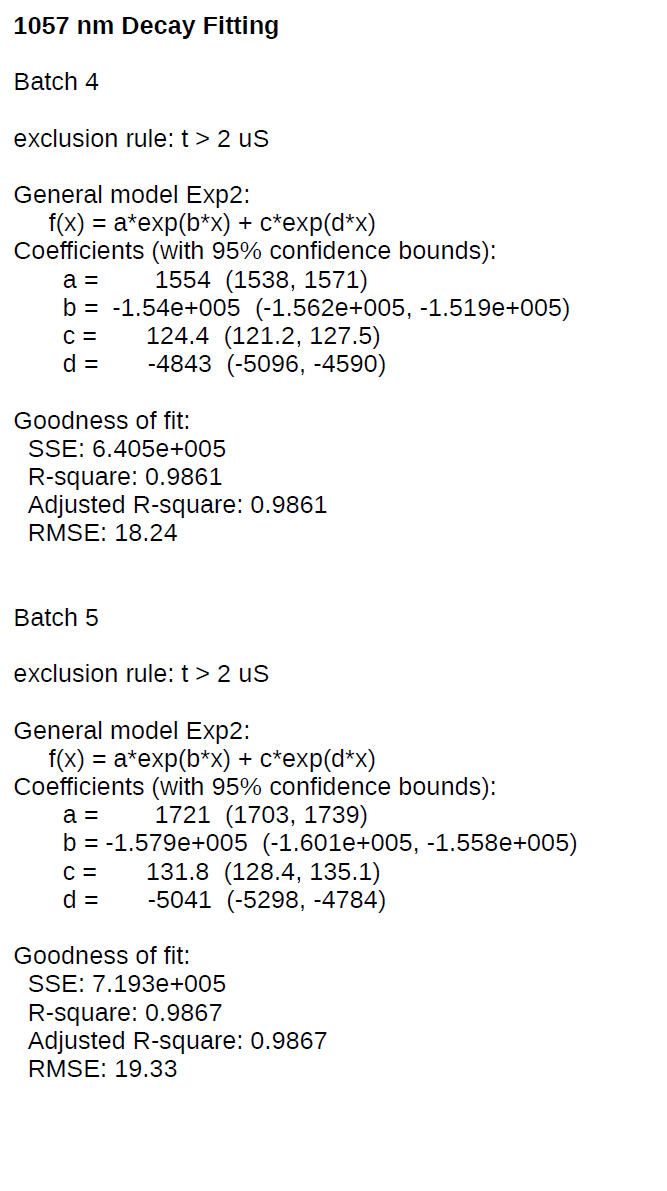

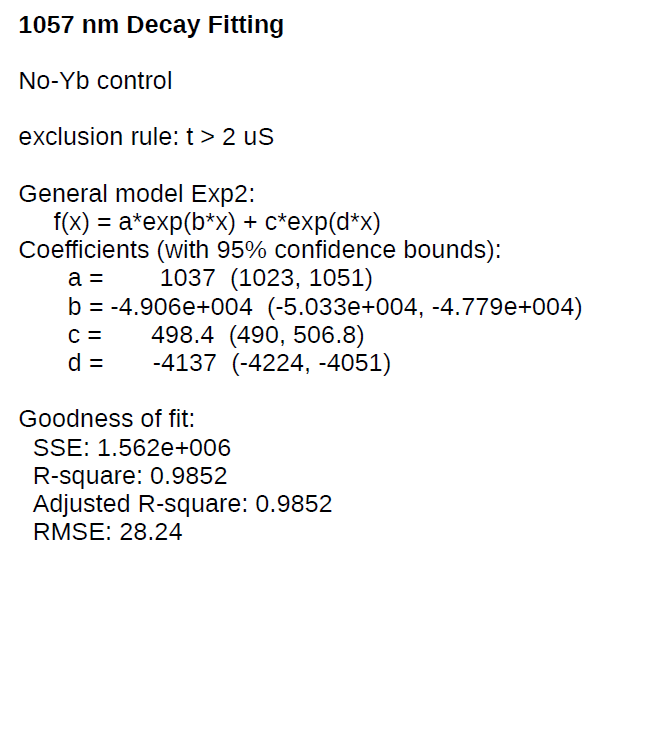


**Figure S8. Spectral Power Dependence - coated nanocrystals featuring Nd^3+^ and Yb^3+^ doping levels of 3% and 20%, respectively.** Data is depicted for three independent batches.

**A)**


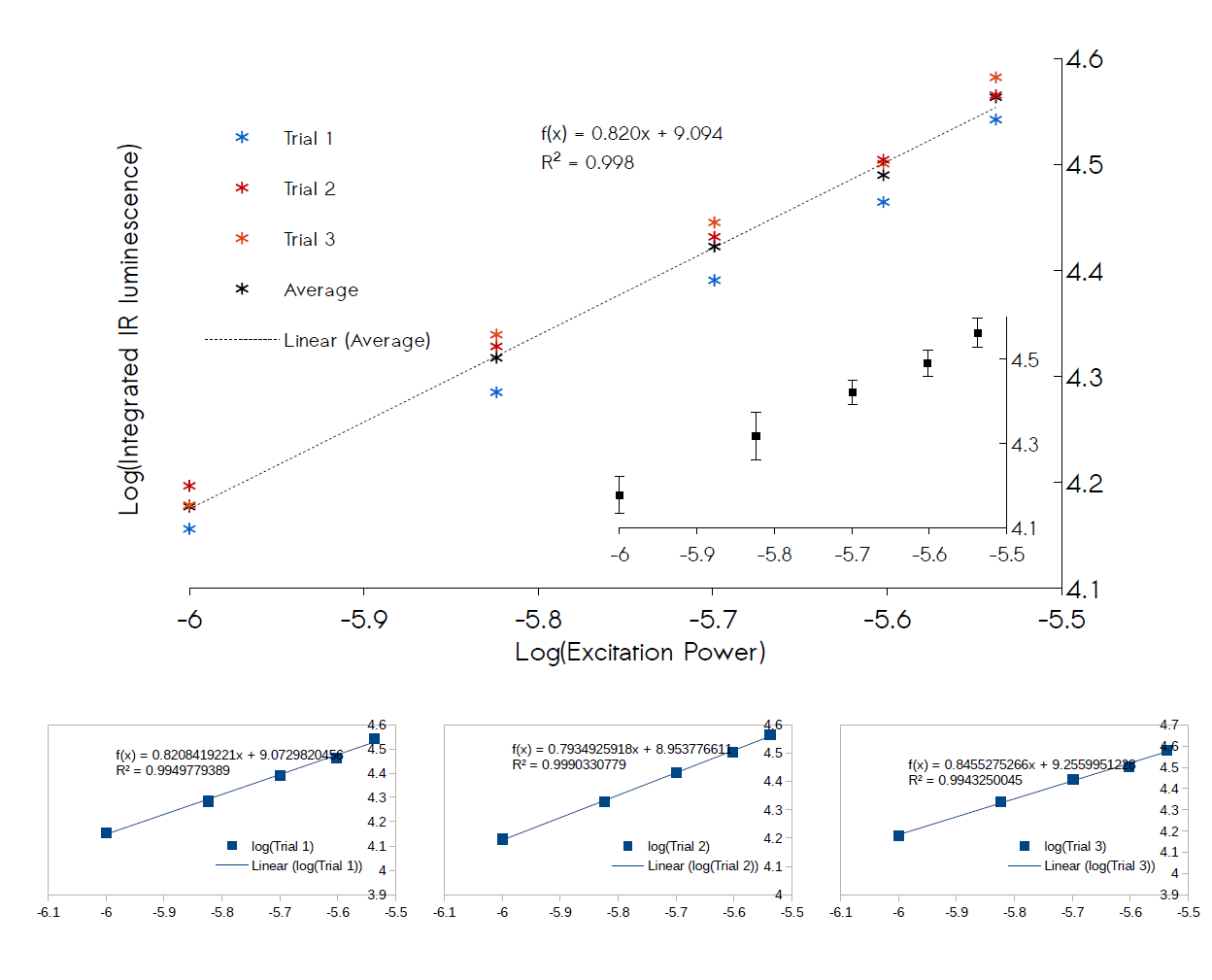

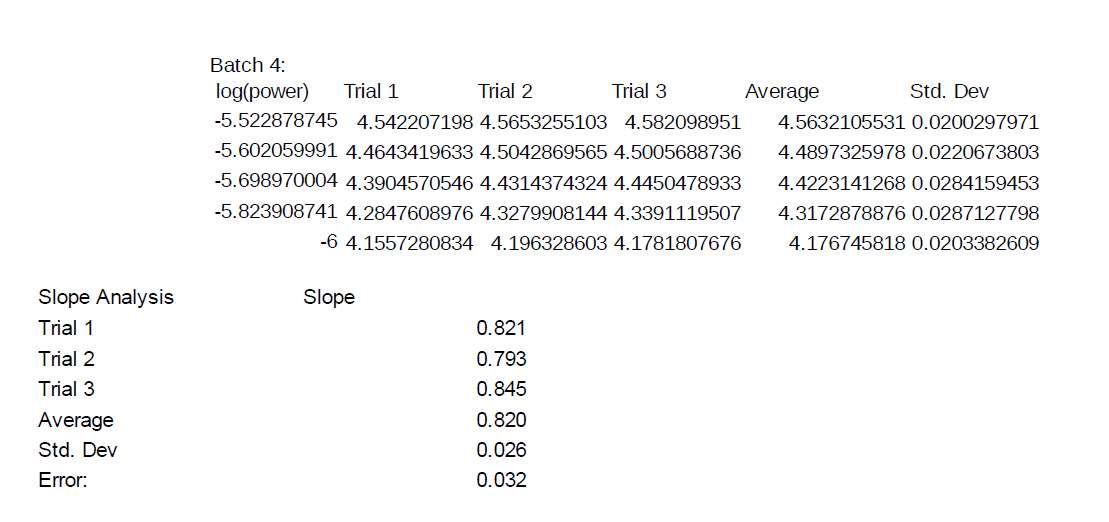


**B)**


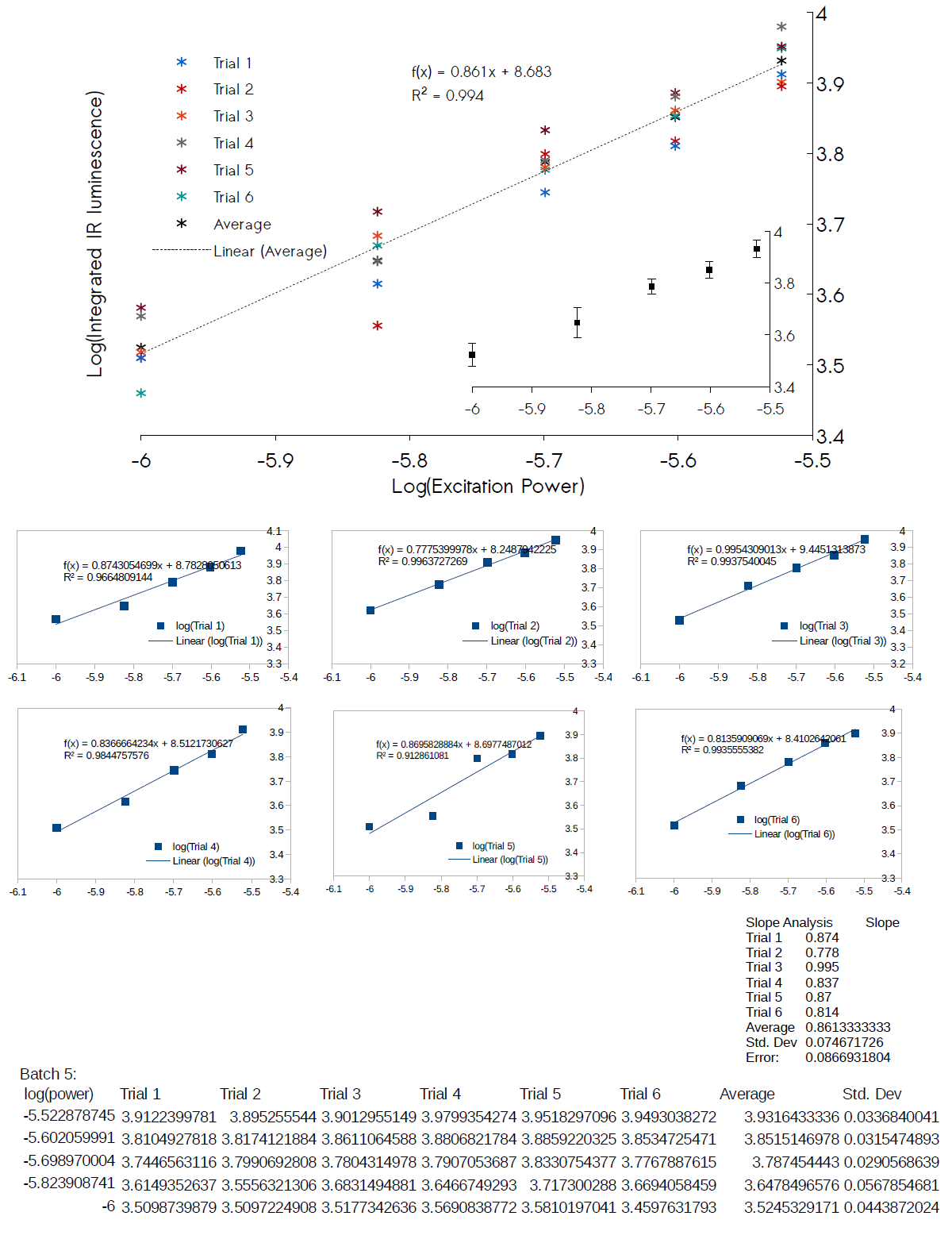


**C)**


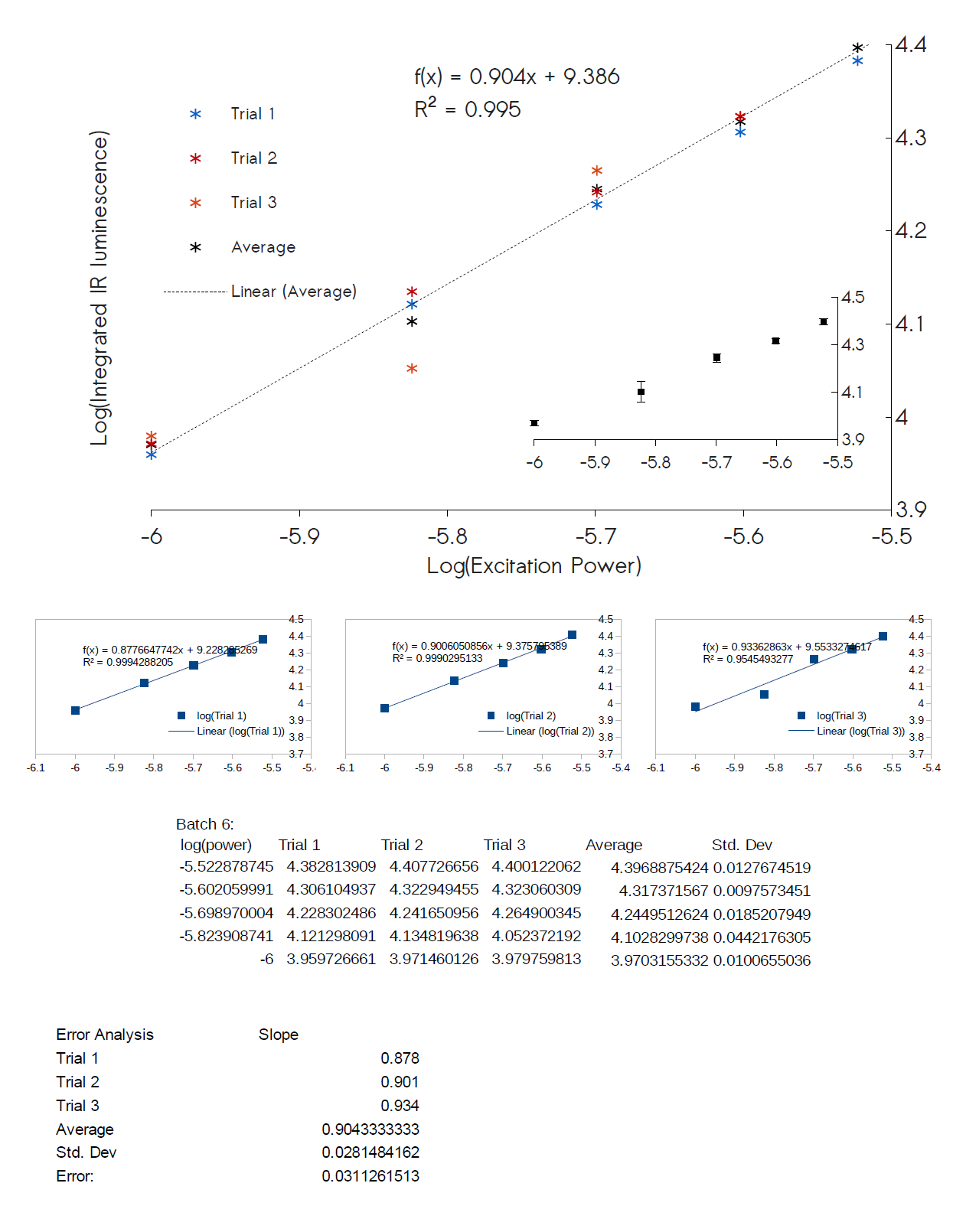


**Figure S9.** **Power Measurement Spectra - coated nanocrystals featuring Nd^3+^ and Yb^3+^ doping levels of 3% and 20%, respectively.** Individual power measurements during ten power dependence trials are reported, along with the raw values of the calculated integrated spectra in the range of 940-1040 nm (Yb^3+^ ^2^F_5/2_ 🡪 ^2^F_7/2_ transition). Data is depicted for three independent batches of nanoparticles (A, B, C), as well as two sets of replicates for batch 6 (B1 and B2).

**A)**


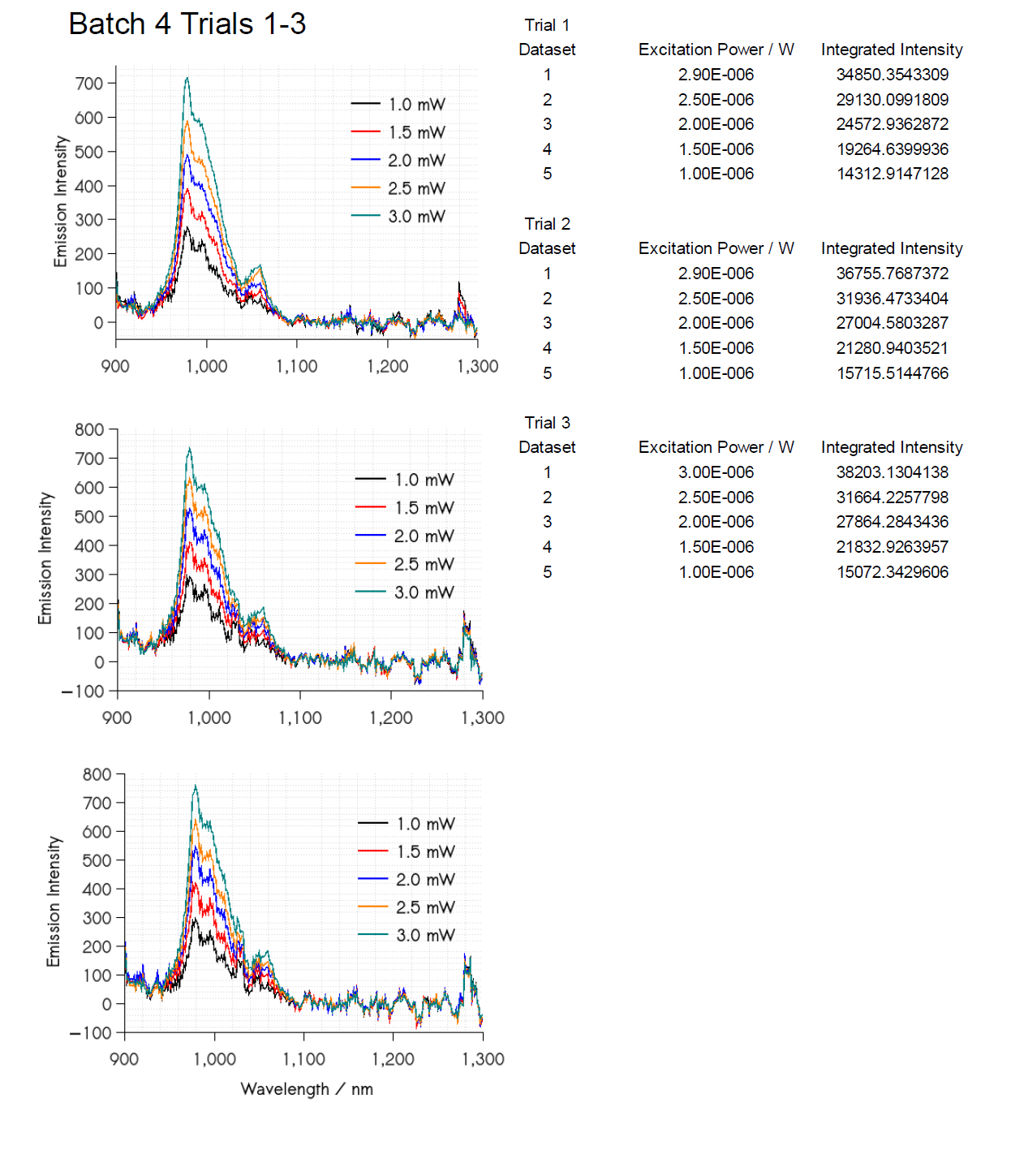


B1)


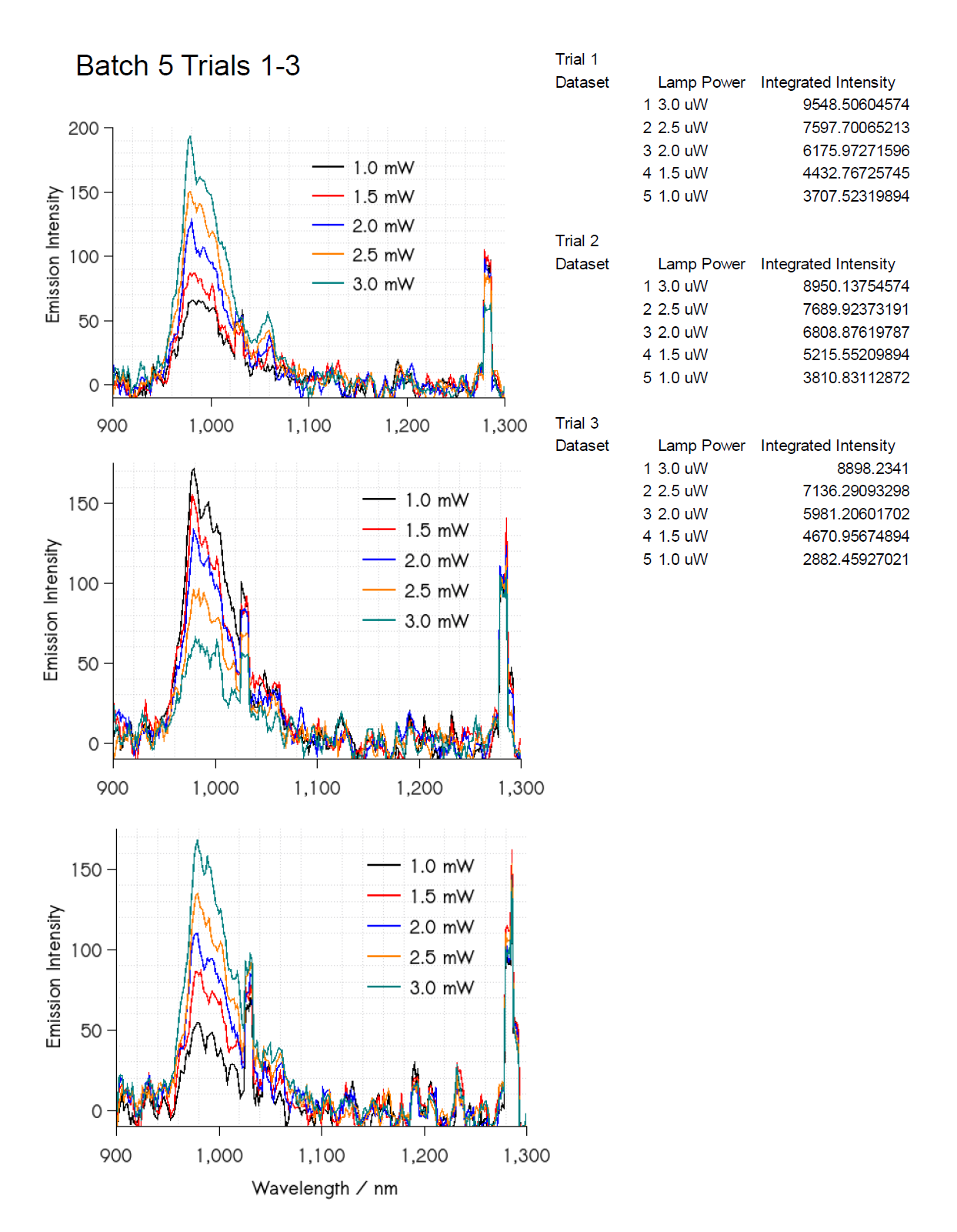


B2)


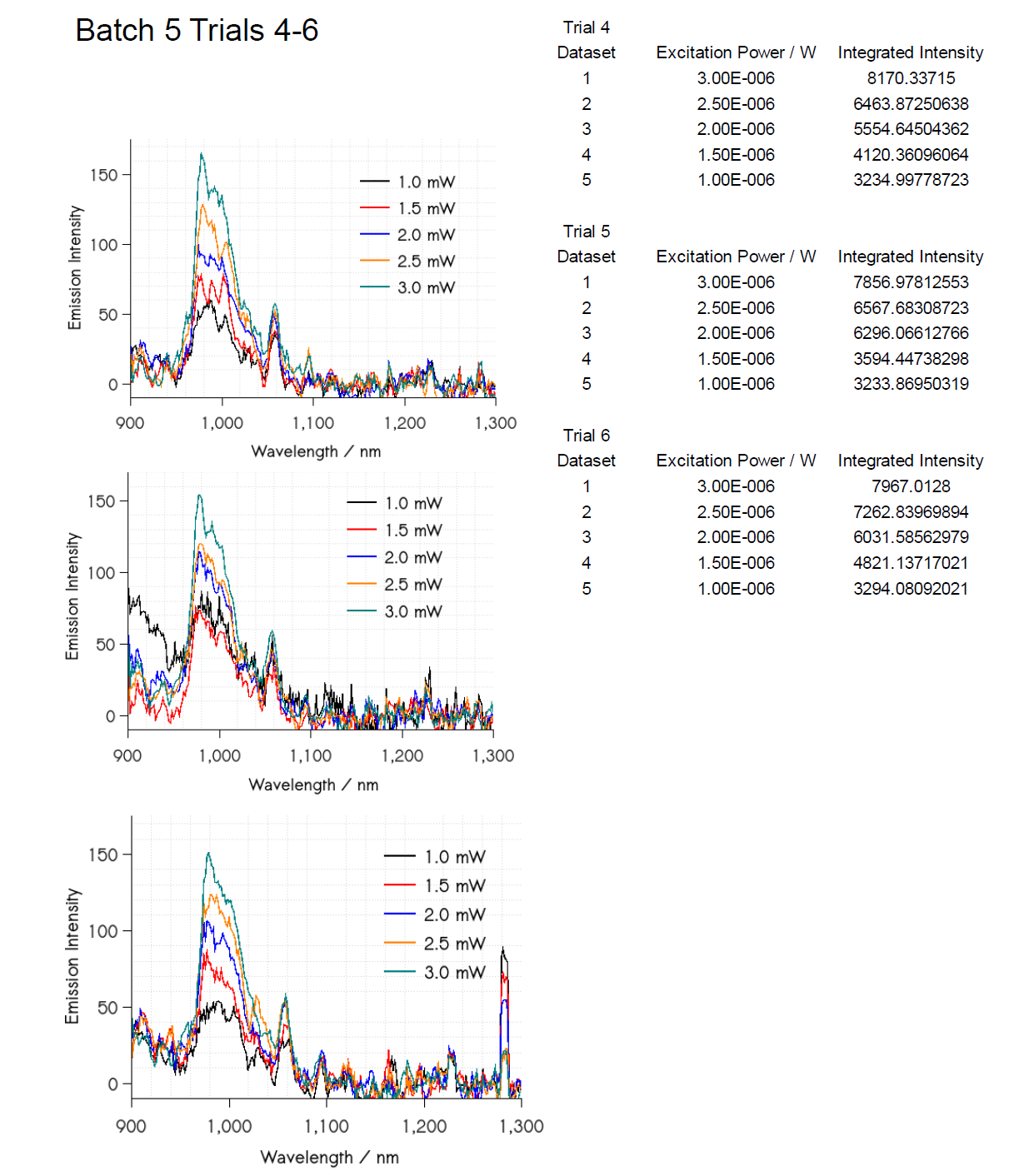


C)


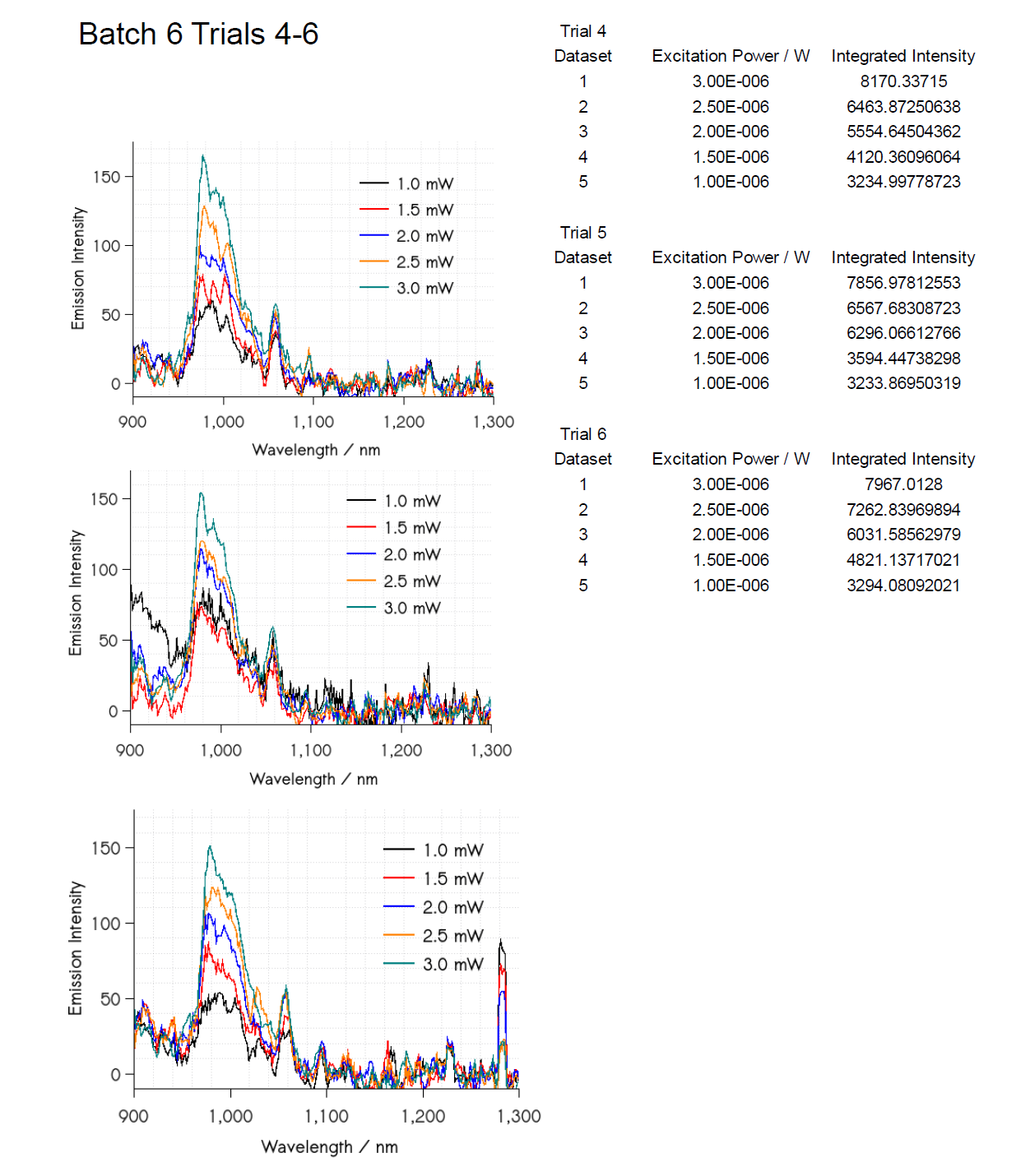


**Figure S10.** **Spectral Power Dependence for Nd^3+^-Only Control NPs.** Data is depicted for two independent batches.

**Figure S11.** Power Density Determination. Data collected at 350-nm excitation, 14-nm bandpass. Data for Neutral Density filter at ~100% transmittance.


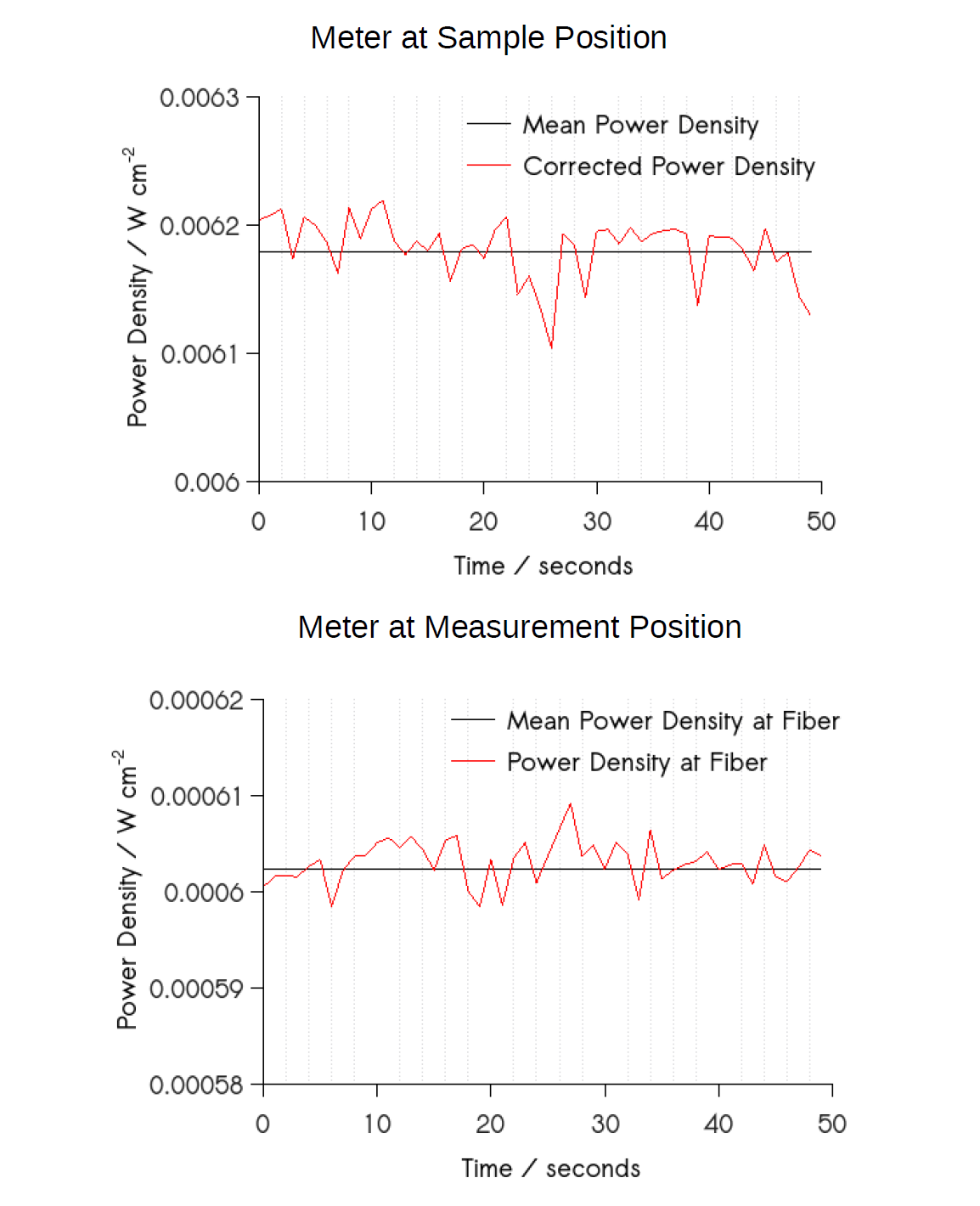


**References**

(1) F. Wang, R. Deng, X. Liu, *Nat, Prot.* **2014**, *9*, 1634.

(2) (a) P. Agbo, R. J. Abergel, *Inorg. Chem*. **2016**, *55*, 9973-9980; (b) P. Agbo, T. Xu, M. Sturzbecher-Hoehne, R. J. Abergel, *ACS Photonics* **2016**, *3*, 547-552.

(3) W. Stręk, A. Bednarkiewicz, P. Dereń, *J. Luminesc.* **2001**, *92*, 229-235.

(4) (a) L. J. Charbonnière, J.-L. Rehspringer, R. Ziessel, Y. Zimmermann, *New J. Chem.* **2008**, *32*, 1055-1059; (b) J. Chen, Q. Meng, P. S. May, M. T. Berry, C. Lin, *J. Phys. Chem. C* **2013**, *117*, 5953-5962; (c) A. M. Cross, P. S. May, F. C. v. Veggel, M. T. Berry, *J. Phys. Chem. C* **2010**, *114*, 14740-14747; (d) J. Goetz, A. Nonat, A. Diallo, M. Sy, I. Sera, A. Lecointre, C. Lefevre, C. F. Chan, K. L. Wong, L. J. Charbonnière, *ChemPlusChem* **2016**, *81*, 526-534; (e) M. Irfanullah, D. K. Sharma, R. Chulliyil, A. Chowdhury, *Dalton Trans.* **2015**, *44*, 3082-3091; (f) S. Li, Z. Hou, Z. Cheng, H. Lian, C. Li, J. Lin, *RSC Adv.* **2013**, *3*, 5491-5497; (g) S. Li, X. Li, Y. Jiang, Z. Hou, Z. Cheng, P. Ma, C. Li, J. Lin, *RSC Adv.* **2014**, *4*, 55100-55107.

**Author Contributions**

P.A. and R.J.A. designed experiments, performed data analysis, and assembled the manuscript; P.A. performed all experimental work; J.S.K. participated in data analysis and quantum yield measurements.
